# Catalyst-Transfer Macrocyclization Protocol: Synthesis of π-Conjugated Azaparacyclophanes Made Easy

**DOI:** 10.1021/jacsau.5c00109

**Published:** 2025-03-07

**Authors:** Josue Ayuso-Carrillo, Davide Bonifazi

**Affiliations:** Institute of Organic Chemistry, University of Vienna. Währinger Strasse 38, 1090, Vienna, Austria

**Keywords:** azaparacyclophanes, catalyst-transfer, macrocyclization, triarylamines, Buchwald−Hartwig, cross-coupling

## Abstract

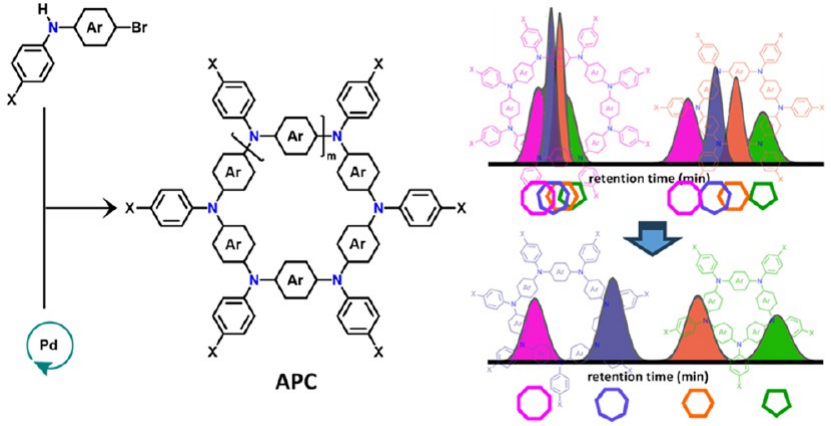

The present Protocol describes the application of the catalyst-transfer macrocyclization (CTM) reaction, focusing on the synthesis of aza[1_n_]paracyclophanes (APCs). APCs are fully π-conjugated shape-persistent macrocycles with potential supramolecular chemistry and materials science applications. This method leverages the Pd-catalyzed Buchwald–Hartwig cross-coupling reaction to selectively form π-conjugated cyclic structures, a significant advancement due to its efficiency, versatility, and scalability. Overall, this Article highlights the following attributes of the CTM method: a) Efficiency and Yield: The CTM method works at mild temperatures (40 °C) and short reaction times (≥2 h), producing high yields of APCs (>75% macrocycles). It avoids the typical high-dilution conditions, making it more practical for large-scale applications. b) Versatility: The method allows the synthesis of APCs with diverse endocyclic and exocyclic functionalities and ring sizes (typically from 4- to 9-membered rings), expanding the chemical space for these compounds. This flexibility is crucial for tailoring APC properties for specific applications. c) Scalability and Reproducibility: Unlike many macrocyclization reactions, which require highly dilute conditions, CTM can perform under concentrated regimes (35–350 mM), making it more suitable for large-scale applications. d) Applications in Materials Science: APCs are noted for their potential in optoelectronic applications due to their π-conjugated structures, which are helpful in organic semiconductors, light-harvesting systems, and other advanced materials. This approach addresses the challenge of complicated multistep syntheses that have hindered the widespread integration of APCs into functional devices. A step-by-step guide to preparing exemplary APCs, including troubleshooting, is provided with photographic illustrations.

## 1 Introduction

**Scheme 1:**
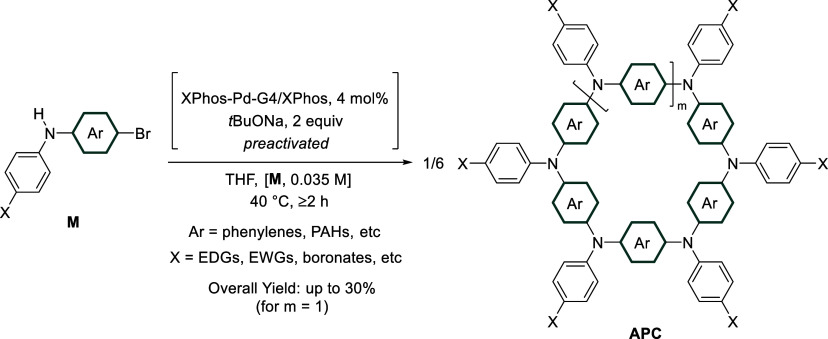
General One-Step Catalyst-Transfer Macrocyclization (CTM) Reaction to Form π-Conjugated Azaparacyclophanes (APCs)

π-Conjugated macrocycles encompass an important class of molecular species that have recently received significant attention due to their prototypical applications in fields like supramolecular chemistry,^1−4^ e.g., molecular/ionic recognition, sensing, molecular separation and catalysis, and materials science,^5−9^ e.g., organic electronics, optics, and light-harvesting systems. These macrocycles are ring-like macromolecules that exhibit fully shape-persistent topologies with inherent rigid, noncollapsible backbone possessing a lumen, i.e., cavity, whose diameter can range from one to several nanometers.^10^ Aza[1_n_]paracyclophanes (APCs) are a prime exemplar category of π-conjugated macrocycles possessing highly symmetric and aesthetic triarylamine-based structures.^11,12^ The bulky propeller-shaped triarylamine (TAA) unit has been positioned as a privileged motif in numerous hole-transport materials (HTM, hole mobilities of 10^–2^–10^–5^ cm^2^ V^–1^ s^–1^) with applications in organic light-emitting diodes,^13−15^ solar cells,^16−19^ field-effect transistors,^20−22^ and electrochromic displays.^23−25^ Typically, APCs display stable radical cations upon electrical stimulus and distinctive color changes upon reversible electrochemical bias.^11,26^ Based on electrochemical measurements and the number of N atoms, up to six reversible oxidations (forming the hexacation) were reported for six-membered ring APCs.^27,28^ Before our one-step report,^27^ a handful of six-membered ring APC examples were reported, all synthesized via multistep approaches and harsh reaction conditions.^28−35^ In this Protocol, we present a detailed description of the synthetic procedure of APCs via the one-step catalyst-transfer macrocyclization (CTM) method (Scheme 1). Both synthetic alternatives are depicted using a glovebox or under standard laboratory conditions. Purification of the APC bulk material product and their further separation into their constituent sizes are also described. By providing this step-by-step protocol, the widespread adoption of the CTM method to prepare novel APC materials is intended.

## 2 Materials

**Figure 1:**
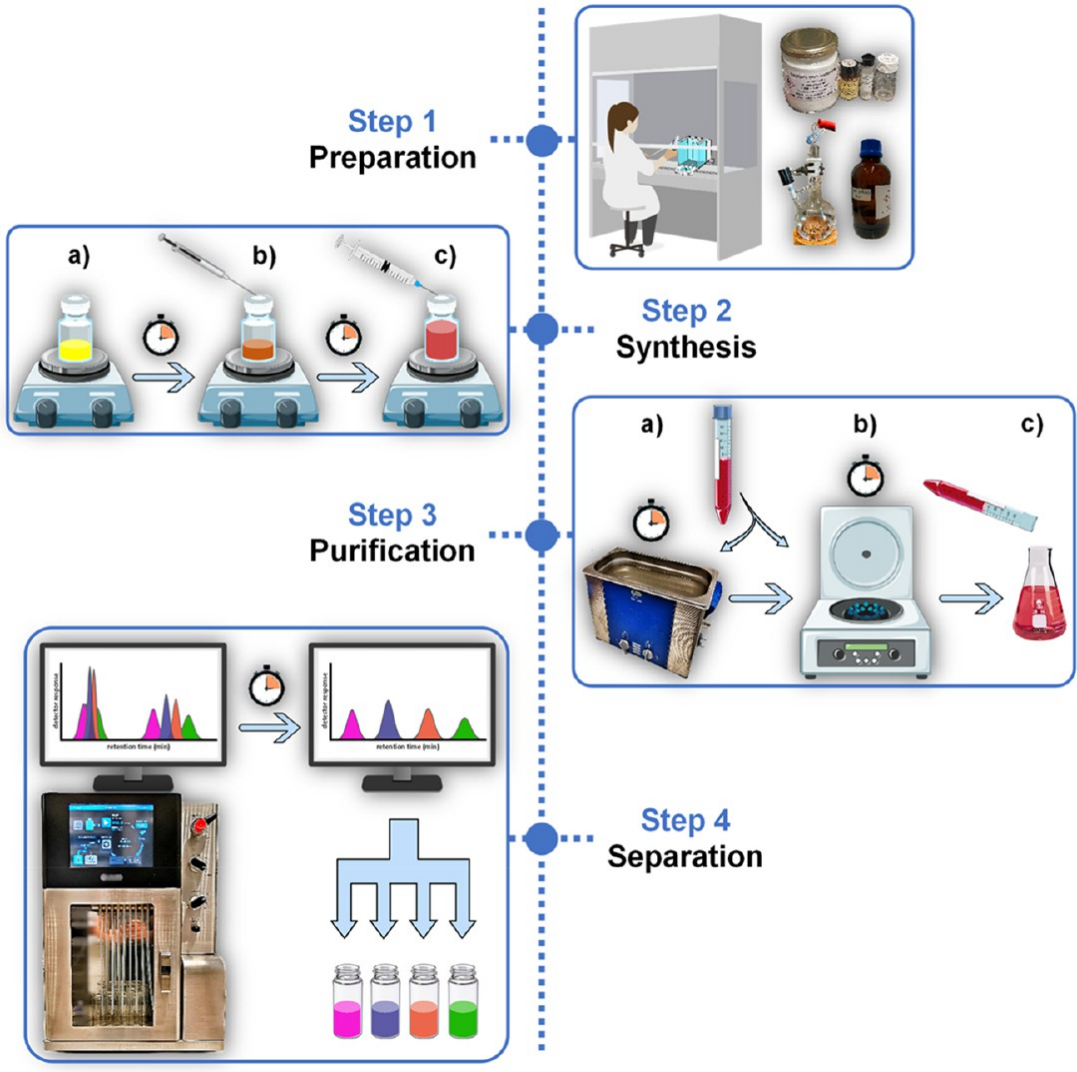
Protocol steps to carry out a Catalyst-Transfer Macrocyclization (CTM) reaction.

Common reagents were sourced from commercial vendors (Table 1). No difference was observed in the reaction outcomes when purchased or freshly prepared^36^ Buchwald G4 palladacycles, such as XPhos-Pd-G4 (methanesulfonato(2-dicyclohexylphosphino-2′,4′,6′-tri-i-propyl-1,1′-biphenyl)(2′-methylamino-1,1′-biphenyl-2-yl)palladium(II)), were used. In general, standard laboratory equipment was employed (Table 2–4), except for the final Step 4: Separation, which requires a preparative GPC instrument (Figure 1). Other instrumentation needed: Access to analytical services such as MALDI-TOF mass spectrometry and NMR spectroscopy.

**Table 1:** Key Reagents

**Reagent**	**Source**	**CAS number**
	**Synthesis**	
XPhos-Pd-G4	Strem/Sigma-Aldrich	1599466-81-5
XPhos	Strem/BLDPharm	564483-18-7
*t*BuONa	Acros Organics	865-48-5
Monomer	Synthesized	---
THF	Anhydrous, degassed, stabilizer-free	109-99-9
**Purification**		
MeOH/HCl 1N (1:1 vol) solution	Prepared from MeOH (Honeywell) and HCl 36% (Fisher Scientific)	---
MeOH	Honeywell (HPLC grade)	67-56-1
Water	Distilled	7732-18-5
**Separation**		
Toluene	Sigma-Aldrich (HPLC grade)	108-88-3

**Table 2:** Equipment (Glovebox Experiments)

**Resource**	**Source**	**Model**
Weighing balance	Ohaus	PR series (0.1 mg)
Magnetic stir hot plate	Heidolph	MR Hei-Tec
EPA vials with PTFE/Silicone septa and cap	Fisher Scientific	20 mL
Teflon-coated magnetic stirrer bars	Fisher Scientific	8 × 3 mm cylindrical with pivot ring
Spatula	Bochem	various
Syringes	B. Braun/Henke Sass Wolf	1 mL/2 mL, 5 mL, 10 mL
Needles	B. Braun	0.80 × 120 mm 21G, 0.6 × 80 mm 23G, 0.9 × 70 mm 20G, 0.8 × 40 mm 21G
Heating block (aluminum)	University Mechanical Workshop	Custom-made
Diamond-shaped weighing boats	Sigma-Aldrich	5 mL, antistatic
Antistatic gun	Zerostat	Milty Zerostat 3
Gas-tight microliter syringes	Hamilton	100 μL
Precision wipes	Kimberly-Clark Professional	Kimtech Science
Argon-filled glovebox with freezer (−39 °C)	MBraun	Labstar

**Table 3:** Equipment (Standard Laboratory Conditions Experiments)

**Resource**	**Source**	**Model**
Weighing balance	Sartorious	A210P (0.1 mg)
Magnetic stir hot plate	Heidolph	MR Hei-Tec
EPA vials with PTFE/Silicone septa and cap	Fisher Scientific	20 mL
Teflon-coated magnetic stirrer bars	Fisher Scientific	8 × 3 mm cylindrical with pivot ring
Laboratory oven	Heraeus Instruments	Kelvitron
Syringes	B. Braun/Henke Sass Wolf	1 mL/2 mL, 5 mL, 10 mL
Needles	B. Braun	0.80 × 120 mm 21G, 0.6 × 80 mm 23G, 0.9 × 70 mm 20G, 0.8 × 40 mm 21G
Desiccator (PO_5_-filled)	Schott	Various
Gas-tight microliter syringes	Hamilton	100 μL
Spatula	Bochem	various
Heated silicon oil bath	Neuber’s Enkel Gross-Drogerie	Silikonöl AK 350
GC vial with screw-caps PTFE/silicone septa	VWR	1.5 mL
Vacuum manifold attached to a high-vacuum pump, regulated supply of argon and a mercury-filled bubbler	University Glassblowing Workshop/Edwards	Custom-made Schlenk line with 5-port PTFE valves/RV12 vac pump

**Table 4:** Equipment (for APC Purification and Separation)

**Resource**	**Source**	**Model**
Rotary evaporator	Heidolph	Hei-VAP Expert
Centrifuge tubes	Fisher Scientific	Polypropylene, 15 mL, plug seal cap
Ultrasound bath	Elma	Elmasonic S 60 H
Centrifuge	Hettich	Universal 320
Vials	Fisher Scientific	50 mL
Syringe filter	Fisher Scientific	PTFE membrane 0.2 μm
Gas-tight syringe	Hamilton	10 mL, 1010 TLL blunt needle
Preparative Recycling GPC	Japan Analytical Industry Co., Ltd.	LaboACE LC-7080, with four UV detectors (265–700 nm)
SEC columns	Japan Analytical Industry Co., Ltd.	JAIGEL-2HR/JAIGEL-2.5HR

## 3 Procedure

The following section describes the sequential stages required to synthesize and purify representative examples of APCs (see 4. Expected Results, Examples 1–3). Practical guidance and advice for preparing small molecules via Pd-catalyzed amination^37^ and conjugated macromolecules^38^ reported elsewhere can complement this Protocol. An overview of the protocol steps used to prepare APCs via a CTM reaction is depicted in Figure 1.

### Step 1: Preparation

Ensure that all necessary laboratory gear, reagents, and solvents are ready to start. Glassware should be dried in a laboratory oven overnight (*T* ≥ 120 °C) and allowed to cool down to ambient temperature inside a desiccator or under vacuum (if attached to a Schlenk line). Reagents in anhydrous form must be employed to ensure reproducibility. For instance, commercial *t*BuONa should be finely ground, heated at ∼120 °C under vacuum, and stored under an inert atmosphere. Synthesized aniline monomers must be kept under an inert atmosphere after the last purification. Buchwald palladacycles^36,39^ and phosphine ligands^40−42^ are air-stable,^43^ but it is advised to keep them in an inert atmosphere for indefinite storage. Anhydrous and dioxygen-free solvents must be procured, which can be achieved via fresh collection from a solvent purification system (SPS)^44^ or distillation from Na/benzophenone ketyl (for THF).^45^ Commercial dry solvents with sure-seal caps can be used, provided they are stabilizer-free.^46^ Freshly collected solvents can be stored in Straus flasks under molecular sieves^47^ (3 Å, followed by ×3 freeze–pump–thaw cycles) for direct use in the glovebox or connected to a Schlenk line via cannula or syringe.

### Step 2: Synthesis (CTM)

#### Estimated Time: 3–24 h

**Figure 2:**
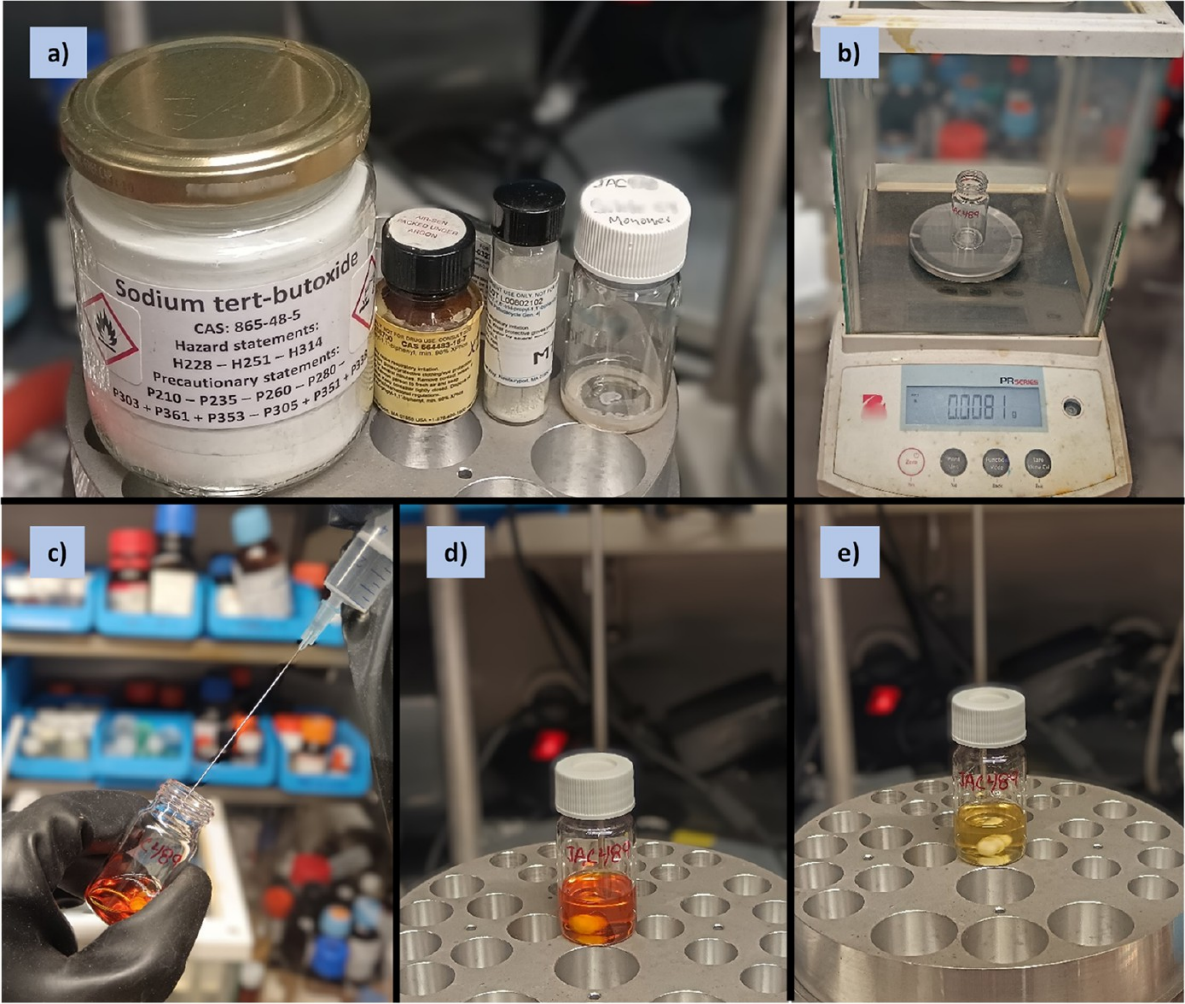
Photographs series for the synthesis of APCs (**1**) performed under a controlled inert atmosphere (Example 1, inside a glovebox): a) reagents; b) weighing of precatalyst, ligand, and base in EPA vial; c) addition of THF; d) precatalyst mixture at the onset of activation; e) catalyst mixture after activation (1 h at 40 °C).

a) Precatalyst activation. The precatalyst/ligand and base are measured (4 mol % and 2.1 equiv relative to monomer, respectively) and loaded in the appropriate reaction vessel (Figures 2a–b, 7a). A magnetic stirrer bar and solvent are added, the vessel is closed, and the reaction mixture is stirred at 40 °C for 1 h (Figures 2c–e, 7b–e).

**Pause**: Note the color changes in the reaction mixture at this stage. Usually, there is a dark and clear coloration at the onset of the reaction, which gradually fades until a clear light-yellow color is reached (Figures 2d–e, 7d–e). These physical appearance changes serve as visual indicators of the reaction completion. Alternatively, the Pd^0^ generation at this temperature, completed at >30 min, can be monitored via ^1^H and ^31^P NMR spectroscopy.

**Figure 3:**
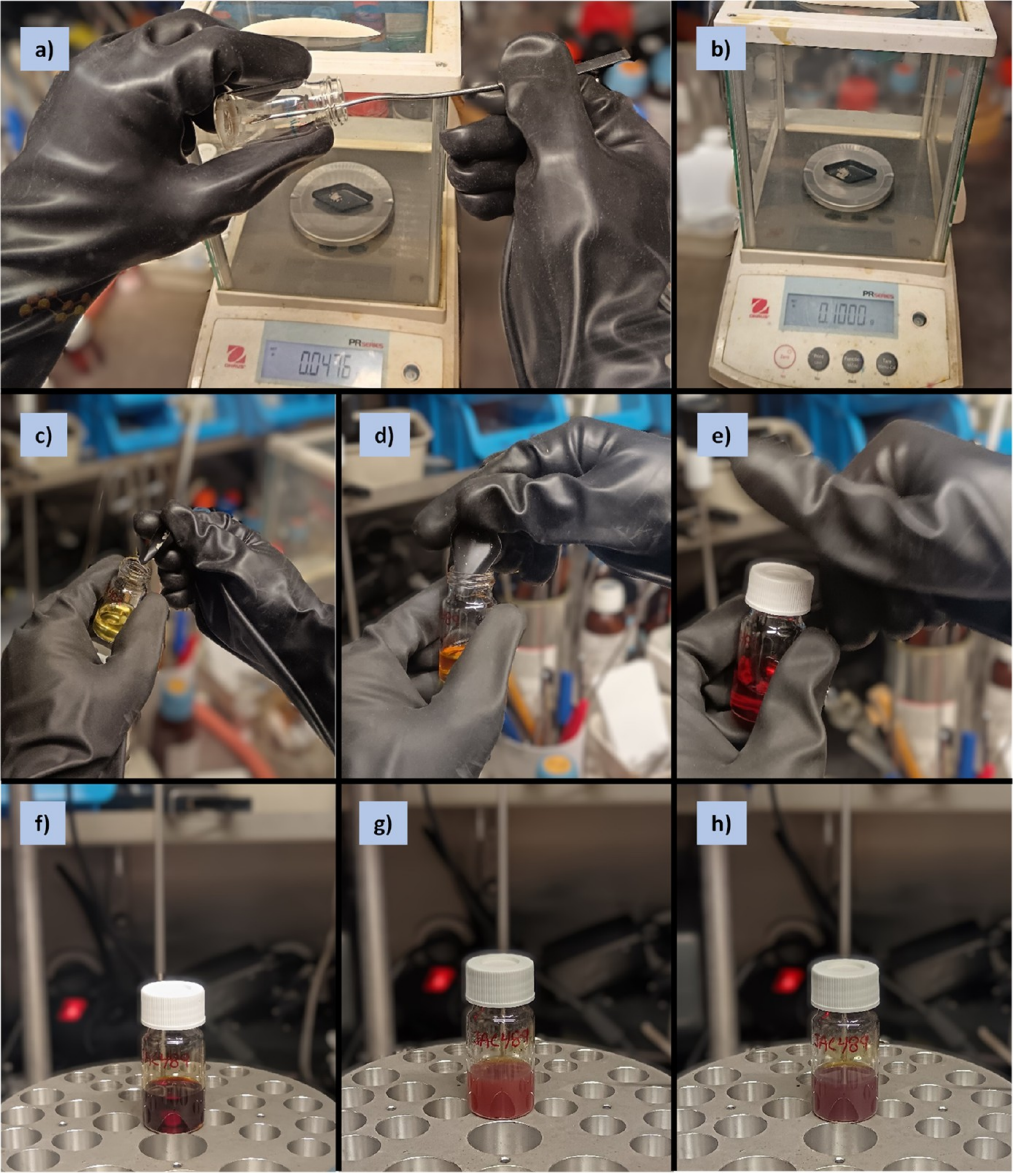
Photographs series for the synthesis of APCs (**1**) performed under a controlled inert atmosphere (Example 1, inside a glovebox): a)–b) weighing of **M1** in a weighing boat; c)–e) sequence of addition of **M1** into catalyst mixture and closure of vial (note solution color change); CTM reaction mixture at f) onset of reaction, g) 1.5 h and h) 4 h (note the color and appearance changes).

b) CTM. The monomer is added to the catalyst solution, the vessel is closed, and the reaction mixture is stirred at 40 °C for >2 h (Figures 3, 7f–h). Liquid monomers can be injected neat via syringe (Figure 7f–g); oil/viscous monomers can be measured and dissolved in a known quantity of solvent before injection via syringe (the volume difference from the precatalyst activation step must be considered to keep the same monomer concentration); solid monomers can be added directly if the inert atmosphere is maintained throughout (Figure 3a–d), or they can be measured into a separate vessel, air evacuated and replaced with inert gas and then dissolved in a known quantity of solvent before injection via syringe (same concentration considerations as above).

**Pause**: Note the color changes of the reaction mixture at this stage. Generally, there is a precise, strong coloration and homogeneous appearance at the onset of the reaction, which gradually fades until a turbid beige-light-yellow color is reached. These physical appearance changes serve as visual indicators of reaction completion (Figures 3c–h, 7f–h). Alternatively, monomer consumption can be monitored via GC MS, NMR spectroscopy, or TLC; APC formation can be monitored via MALDI-TOF MS, analytical GPC, or NMR spectroscopy.

c) Quenching. MeOH/HCl 1N (1:1 vol) solution is added to the reaction mixture to precipitate the product.

**Figure 4:**
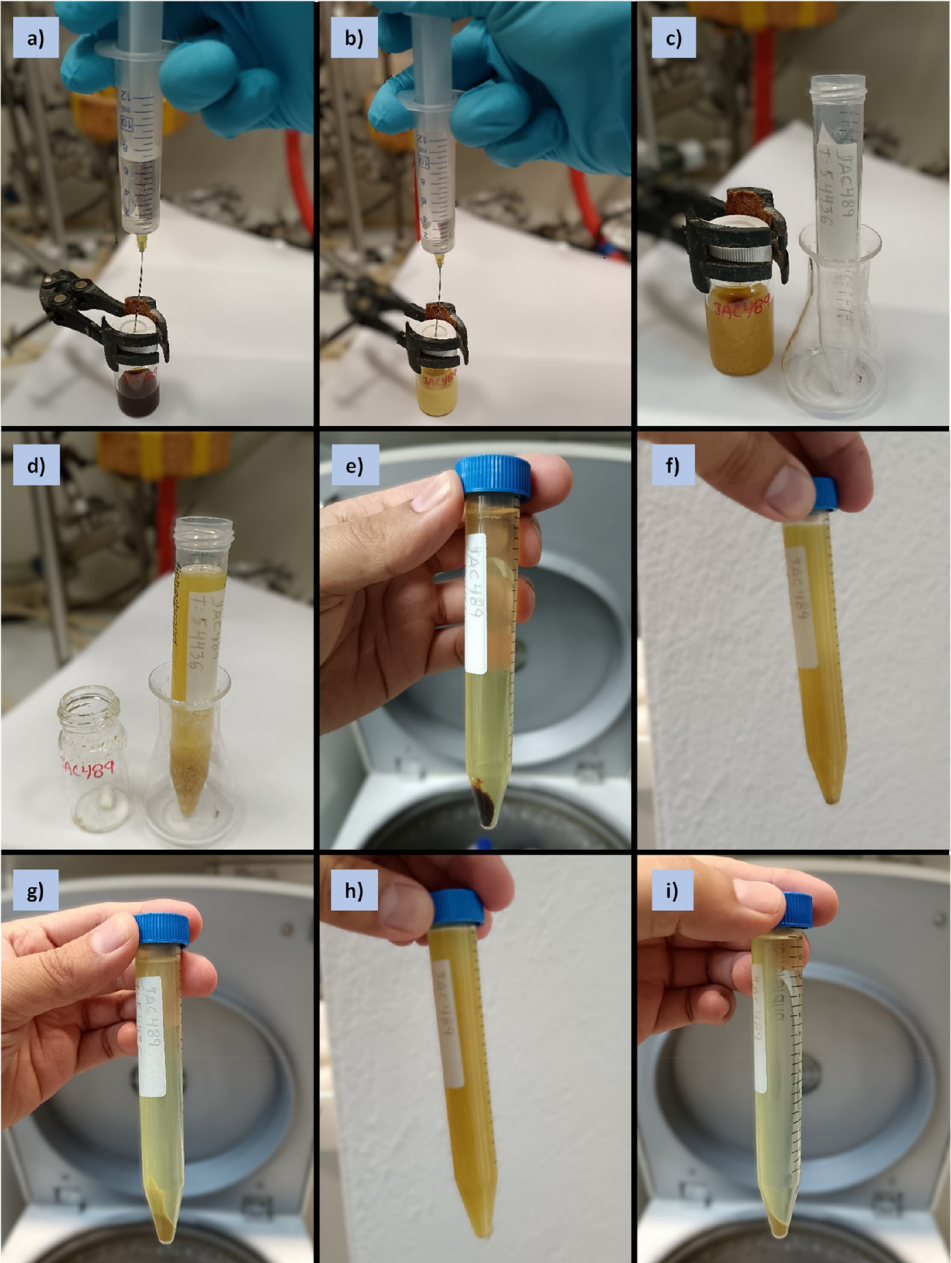
Photographs series for the quenching and purification of APCs bulk materials (**1**, Example 1) performed under standard laboratory settings in air (outside the glovebox): a)–c) sequence of quenching by injecting MeOH/HCl 1N (1:1 vol) into reaction mixture to precipitate the product (note the color and appearance changes); d) transfer of quenched mixture into a centrifuge tube; e) product plus washing solution after centrifugation (first cycle); f) sonication of product dispersed in a mixture of 2 mL distilled water and 12 mL MeOH (second cycle); g) product plus washing solution after centrifugation (second cycle); h) sonication of product dispersed in a mixture of 1 mL distilled water and 13 mL MeOH (third cycle); i) product plus washing solution after centrifugation (third cycle) for further decantation.

**Pause**: Note the appearance changes in the reaction mixture at this stage. Commonly, a dispersion of beige/light-yellow color solids is produced and serves as a visual indicator of successful APC formation (Figures 4a–c, 7i). See Troubleshooting for information on whether dispersed solids are present at this stage (Table 5).

**Table 5:** Troubleshooting Table

**Problem**	**Possible cause**	**Solution**	**Other advice**
**Synthesis**			
Low yields of APC bulk material	Incomplete reaction	Ensure reaction time and temperature are optimal. Check purity of reagents and starting materials	Monitor monomer consumption
	Insufficiently active catalyst	Check purity of precatalyst	Check/monitor precatalyst by ^31^P NMR spectroscopy
	Poor mixing	Check homogeneity of the stirred mixture and ensure proper stirring	Use appropriate stirrer bar/mixer, e.g., with pivot ring
Product not precipitating (at end of reaction)	Inadequate antisolvent (MeOH/HCl) quantity	As a general rule, at least a 1:1 volume ratio of antisolvent/solvent should be used	Reverse the order of addition: add reaction mixture dropwise into stirred antisolvent
	Reaction mixture insufficiently cooled down	Ensure mixture has reached rt at the end of reaction	Store the quenched reaction at ∼5 °C overnight to induce precipitation
**Purification**			
Impurities in isolated APC bulk material	Insufficient washing steps	Repeat washing steps with H_2_O and MeOH	A clear, transparent supernatant solution is expected
	Contaminated reagents, purification solvents or glass-/plasticware	Use reagents and solvents of the highest purity. Ensure glassware is properly cleaned	Use plasticware only once
Redispersion issues	Bulk material stuck at the bottom of the centrifuge tube	Increase sonication time	Tap the tube contents manually to help redispersion
Centrifugation issues	Inadequate centrifugation speed or time	Check speed and duration and increase them if necessary. Verify tube is properly balanced in the instrument	Cool the tube contents down to rt (or below with the aid of an ice bath) if hot after sonication in the previous step
Drying issues	Improper vacuum or freeze-drying conditions	Check for leaks in the vacuum system	We found that the APC bulk material can be air-dried (left open in the fumehood) overnight with no detrimental effects
**Separation**			
Partial bulk material injection into recGPC instrument	Bulk material not fully solubilized	Sonicate with mild heating for ∼5–10 min prior to filtering through 0.2 μm syringe filter	Redesign solubilizing groups of the targeted APC
Individual fractions not pure enough	Insufficient recycling time	Allow longer recycling times	Collect (cut) narrower chromatographic peaks
Diffusion (widening) of the chromatographic peaks over time	Presence of bubble(s) in the loop system	Degas the reservoir solvent prior to separation	Allow longer time for the baseline stabilization prior to separation
	Solvent not optimal for separation	Test a different compatible solvent, e.g., THF, chloroform, etc	Obtain “rough” fractions and repeat the separation procedure

### Step 3: Purification (APC Bulk Material)

#### Estimated Time: 3–8 h

a) Sonication. After reaction quenching (Step 2c), the reaction mixture is transferred into a centrifuge tube. The tube is closed and sonicated at rt for ∼5 min.

**Pause**: Before the next centrifugation step, ensure the dispersed solids have a fine powder appearance (Figure 4).

b) Centrifugation. Subject the product to centrifugation for 15 min at 5000 rpm.

**Pause**: Ensure no solids are suspended in the supernatant liquid phase. Allow longer centrifugation times or higher speeds (Figure 4).

c) Decantation. Decant and discard the liquid phase. Keep the solid product.

**Pause**: Note the liquid phase look. Usually, the liquid coloration fades until it becomes colorless (after >3 purification cycles; see below). These physical appearance changes serve as a rough visual indicator of purification completion. Alternatively, the liquid phase can be analyzed via GC MS or NMR spectroscopy to confirm the absence of byproducts.

e) Washing with antisolvent. The product is dispersed in a mixture of water/MeOH for the next cycle (Step 3a–c). A gradient of water/MeOH is applied (Figure 4); namely, 1:6 (first cycle) → 1:13 (second cycle) → 0:1 volume ratio (third cycle onward).
f) Repeat Steps 3a–e for five times.
g) Drying of APC bulk material. The product is dried under vacuum (10^–3^ mbar for ≥2 h) after the last decantation Step 3c.

**Figure 5:**
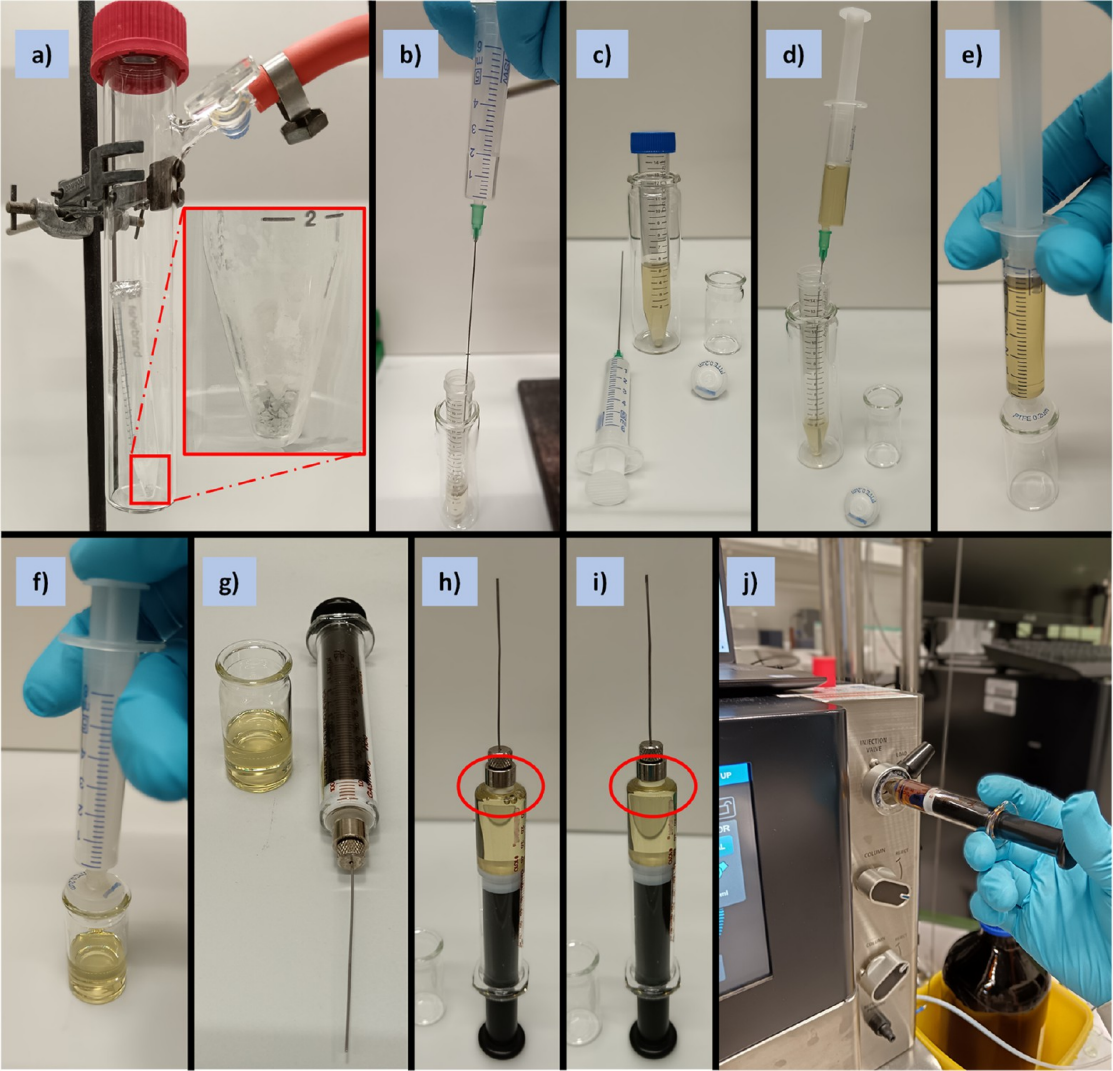
Photographs series for the purification and separation of APCs bulk materials (**1**, Example 1) performed under standard laboratory settings in the air: a) drying of APC bulk material under vacuum (10^–3^ mbar for ≥2 h) after five cycles of sonication/centrifugation/decantation; b)–i) the sequence of preparation of APC bulk material solution in toluene before its j) injection into the recycling GPC instrument for further separation into individual nN ring sizes.

Note: A comparison between this purification procedure and further complementary steps, such as filtering a solution of bulk material through a Celite pad, showed no purity increment at this stage.^27^

**Pause**: Typically, a free-flowing powder is obtained (Figure 5a).

**Figure 6:**
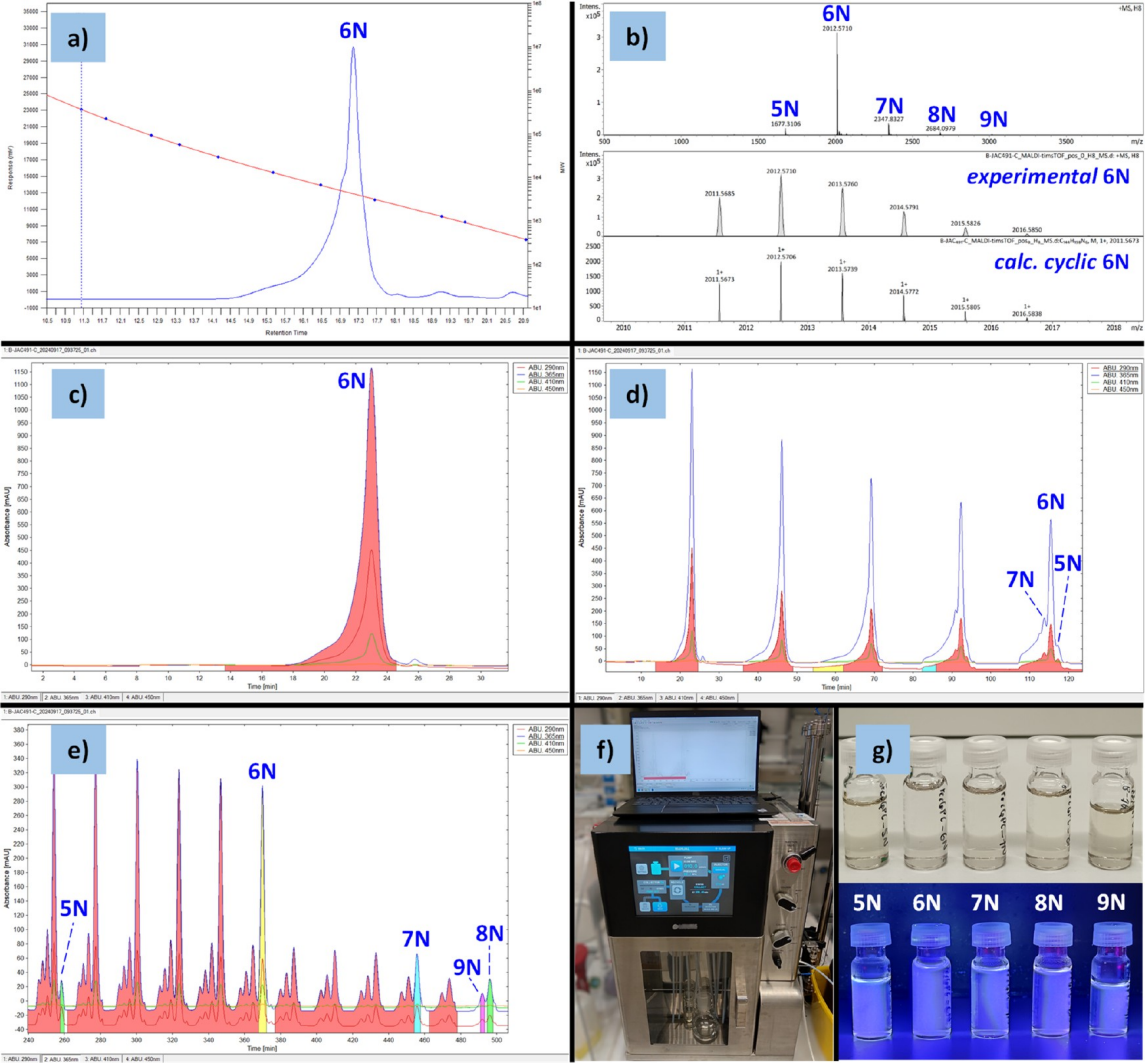
Analysis of the APC bulk material (**1**, Example 1): a) analytical GPC chromatogram, b) HR-MALDI-TOF MS showing the experimental and calculated isotopic patterns for 6N ring (**1**_**6N**_). Preparative recycling GPC chromatograms of the APC bulk material (**1**) during c) the first cycle of the separation stage, d) the first five cycles of the separation stage (∼2 h), e) the elution time window displaying fractions collection (∼240–500 min) with each nN ring sizes indicated. f) Example of fraction collection from the recycling GPC instrument. g) Photographs under ambient light (top) and UV light (bottom, 365 nm) of samples of each isolated nN ring size in THF solution used for both HR-MALDI-TOF MS and analytical GPC analyses.

h) Analysis of APC bulk material. A sample of APC bulk material is analyzed via MALDI-TOF MS, analytical GPC, or NMR spectroscopy to confirm the purity and distribution of APCs. Depending on the endo moiety of the targeted APC, the 6-membered ring species is generally obtained as the major component (Figure 6a–b).

**Critical**: At this stage, while the expected formation of the 6-membered ring APC is anticipated as the major component in the bulk material, other *n*-membered rings can be formed in similar or higher abundance. APCs are highly symmetrical species that share similar chemical environments, which result in similar NMR chemical shifts. Analytical GPC provides a qualitative estimation of the different nN-size distributions in the APC bulk material (where nN refers to the polygon formed with N atoms serving as vertices, e.g., *n* = 5 for the pentagon, etc.). Still, only high-resolution MALDI-TOF MS can unequivocally confirm the APC identity. After multiple repetitions of this reaction with numerous monomers in our laboratory, no linear oligomers were observed, suggesting that complete monomer consumption has been accomplished.

### Step 4: Separation (APC Individual Sizes)

#### Estimated Time: 8–24 h

The following descriptions refer to the preparative recycling GPC instrument operation in the *Manual* mode.

a) Injection of APC bulk material solubilized in toluene into the recycling GPC instrument. Dissolve the APC bulk material in the centrifuge tube in 5 mL of HPLC-grade toluene (Figure 5b–c). Sonicate the solution if necessary to ensure homogeneity. Retrieve the solution with a syringe, remove the needle, and attach a 0.2 μm PTFE membrane syringe filter (Figure 5d–e). Filter the solution into a clean vial (Figure 5f). Take the filtered solution with a gastight syringe (Figure 5i) and inject the solution into the instrument inlet port (Figure 5j, i.e., injection valve, *inject* mode).

**Pause**: Follow the instructions of the instrument’s vendor. Our system’s recommended maximum loading capacity is 100 mg/5 mL solvent (toluene), the maximum flow rate is 15 mL/min, and the maximum pressure is 15 MPa.

Note: Before the injection (Step 4a) ensure the instrument has achieved a stable baseline, which is usually accomplished after ∼20 min of solvent flushing at the set flow rate (e.g., 10 mL/min) and pressure (e.g., ∼ 8 MPa) in *waste* mode.

**Critical**: Ensure no air bubbles are present during the injection into the instrument (Figure 5h–i), as it will destabilize the baseline and might compromise the separation quality.

b) Collection of fractions. Depending on the type and length of columns, pressure, and solvent employed, fractions typically start resolving into individual components after the third GPC recycling cycle. After collection, the solvent of each fraction is removed via rotary evaporation (77 mbar at 40 °C) and dried under vacuum (10^–3^ mbar). Specifically, after Step 4a, the *recycling* mode of the recycling GPC instrument must be turned on as soon as the UV detectors indicate an increase in the absorbance (Figure 6c). Depending on the APC type, this usually occurs within 15–18 min after injection. From this point, careful attention must be paid to the evolution of the peak separation in each cycle. Ideally, each chromatographic peak corresponding to each nN ring size will separate without overlapping with each other. For practical purposes, as soon as any chromatographic peak is sufficiently defined, e.g., with minimum overlapping to their vicinal peaks, its corresponding volume is taken out of the recycling loop, i.e., the solution is collected directly in a round-bottomed flask (Figure 6e–f) by turning on the *collecting* mode of the recycling GPC instrument for the duration of the collection. However, it is advised to discard the volumes corresponding to the front and end tails of this desired fraction, i.e., to get the volume corresponding to the width at half-height of the chromatographic peak. In practice, this procedure is carried out by alternating the *recycling* mode - *waste* mode – *collecting* mode – *waste* mode – *recycling* mode sequence on the instrument. This process is performed until the last fraction is collected.

**Pause**: The chromatogram observed during the first cycle should look very similar to the one obtained from the APC bulk material’s analytical GPC in Step 3h, as it is the same sample, regardless of the detector used in each instrument. For example, compare the chromatogram patterns displayed in Figure 6a (analytical GPC chromatogram, refractive index detector) and Figure 6c (recycling GPC chromatogram, UV detector at 365 nm). After this elution time, the chromatogram peaks will separate into their components over several cycles (Figures 1, 6d–e).

Note: Ensure that the loading loop of the inlet port is closed after injection. If the loop is left open, the retention time for each cycle will be different.

**Critical**: Each class of APC will have different hydrodynamic radii among their produced nN ring sizes, which means that the separation efficiency in peak quality and purity will not necessarily be the same for each APC class when using the same recycling GPC instrument/columns setup. For example, when facing a broad peak fraction that seems particularly difficult to separate even after several cycles, a decision needs to be made: either collect the fraction containing the multiple ring sizes (especially true for ≥9N rings) or leave them cycling for a longer time, e.g., overnight (see Troubleshooting, Table 5). In our experience and for practical purposes, the higher molecular weight fraction that contains large ring sizes and elutes at early times is first collected (Figure 6d, as it is usually a minor component of the bulk material, e.g., 10–20%) to allow the more abundantly formed 5N–9N APCs for better separation.

c) Analysis of each fraction. Analogously to Step 3h, a sample of each collected fraction is analyzed via HR MALDI-TOF MS, analytical GPC, and NMR spectroscopy to confirm each APC’s identification (Figures 6g, 8–9).

**Figure 7:**
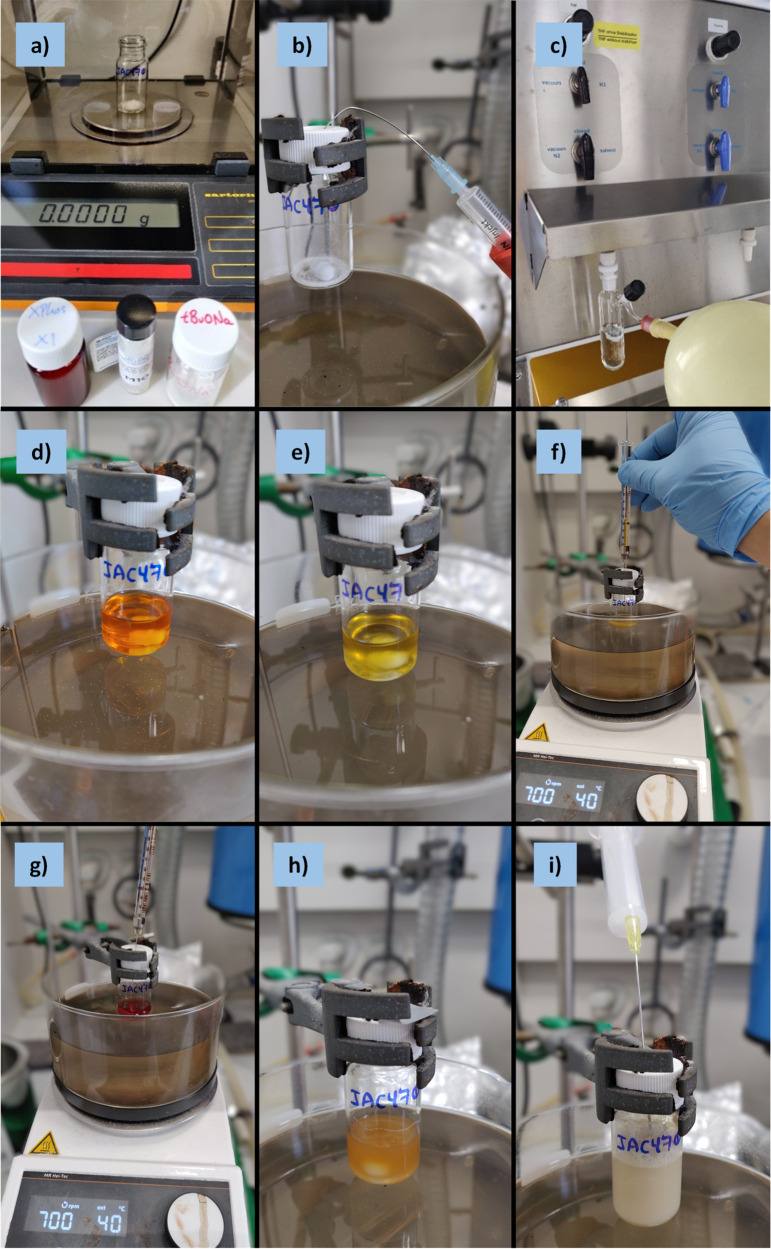
Photographs series of APC (**3**, Example 3) synthesis performed under standard laboratory settings, at the benchtop with the aid of a Schlenk line (outside the glovebox): a) weighing of precatalyst, ligand, and base in EPA vial; b) vessel evacuation/refilling with argon via Schlenk line; c) collection of anhydrous THF from an SPS; d) precatalyst mixture after addition of THF via syringe at the onset of activation; e) catalyst mixture after activation (1 h at 40 °C); f)–g) the sequence of addition of **M3** into catalyst mixture via syringe; h) CTM reaction mixture after 2 h; i) quenching by injecting MeOH/HCl 1N (1:1 vol) into the reaction mixture to precipitate the product.

**Figure 8:**
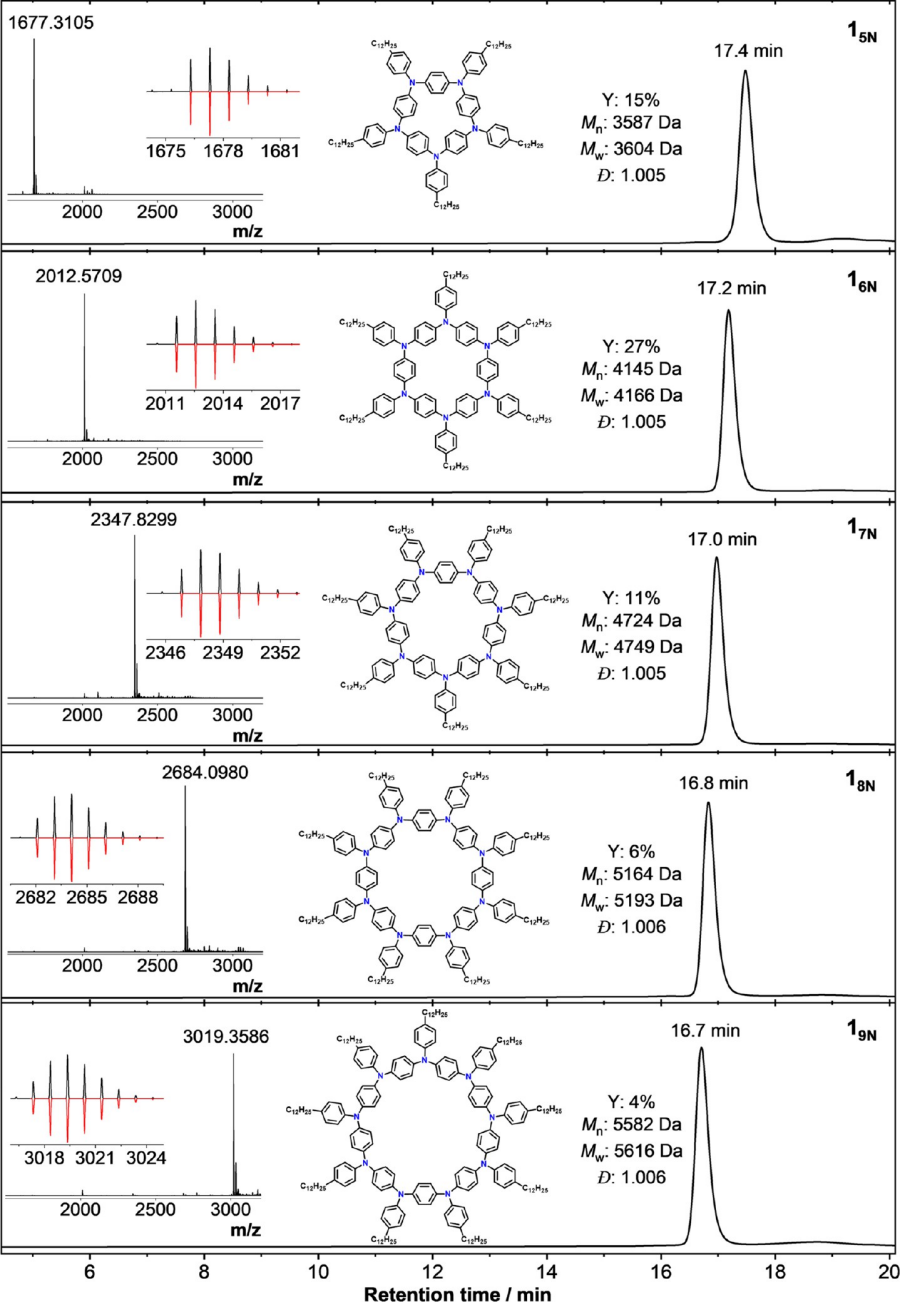
Characterization by analytical GPC of isolated individual **1** (Example 1) macrocycles obtained after subjecting 64 mg of bulk material to preparative recycling GPC, yielding fractions of different nN ring sizes. From top to bottom: **1**_**5N**_, **1**_**6N**_, **1**_**7N**_, **1**_**8N**_, and **1**_**9N**_. Inset: HR-MALDI-TOF MS spectra with their experimental (positive, black) and calculated (negative, red) isotopic patterns. Yield relative to that of starting monomer **M1**. Metrics quoted for GPC analysis based on polystyrene calibration standards.

**Figure 9:**
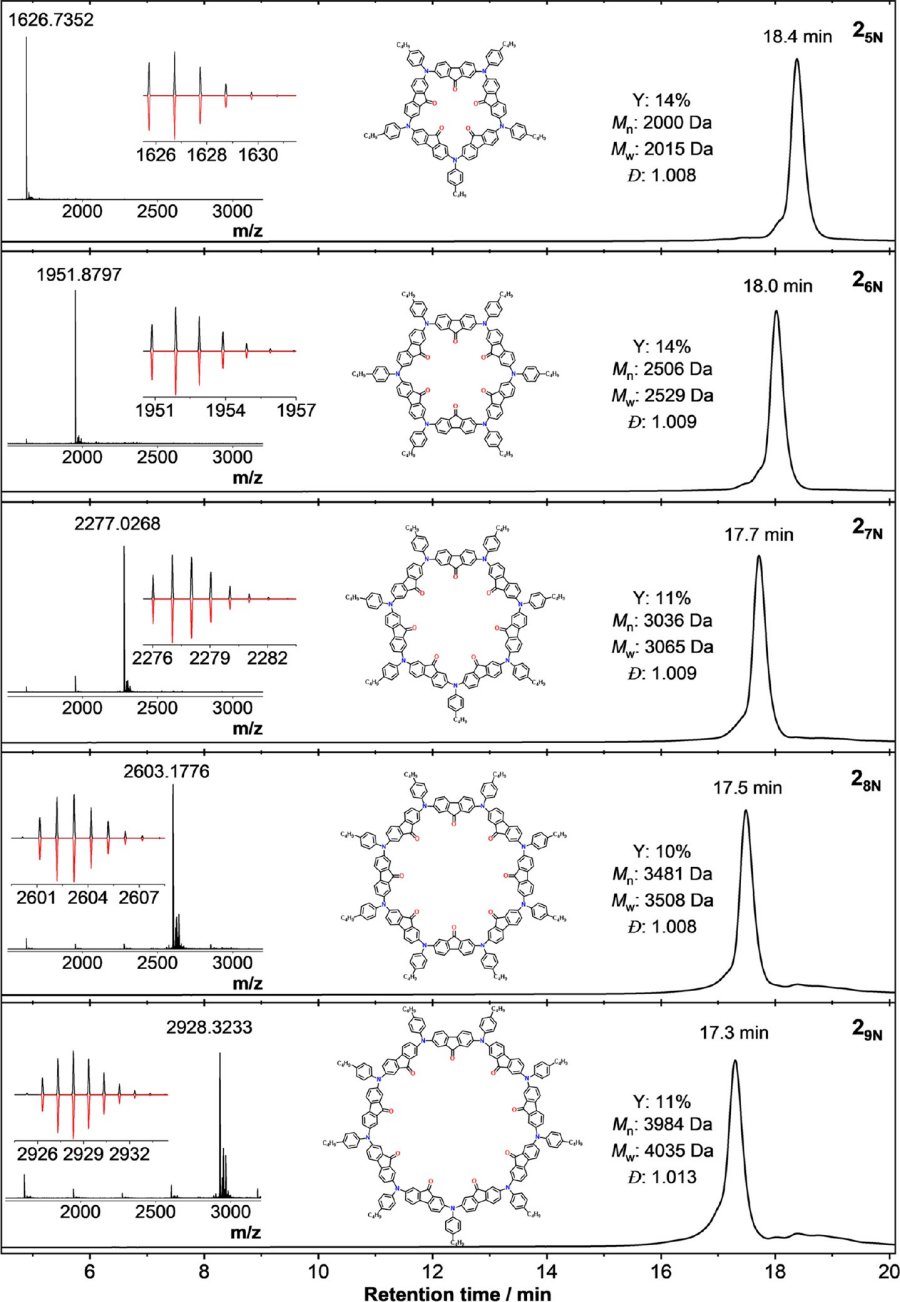
Characterization by analytical GPC of isolated individual **2** (Example 2) macrocycles obtained after subjecting 100 mg of bulk material to preparative recycling GPC, yielding fractions of different nN ring sizes. From top to bottom: **2**_**5N**_, **2**_**6N**_, **2**_**7N**_, **2**_**8N**_, and **2**_**9N**_. Inset: HR-MALDI-TOF MS spectra with their experimental (positive, black) and calculated (negative, red) isotopic patterns. Yield relative to that of starting monomer **M2**. Metrics quoted for GPC analysis based on polystyrene calibration standards.

### 3.1 APC Synthesis under the Inert Atmosphere Inside a Glovebox

In general, an argon-filled glovebox (O_2_ and H_2_O levels <1 ppm) was employed throughout our previous report^27^ to ensure reproducibility, mainly because, as it is well-known for this chemistry, Buchwald–Hartwig cross-coupling reactions require anhydrous conditions.^48,49^ Additionally, in our hands, some of the monomers proved to be hygroscopic to some extent, which might detrimentally affect the course of the reaction by, for example, decreasing the yields of the bulk materials. Refer to examples 1 and 2 for a detailed description.

### 3.2 APC Synthesis under Standard Laboratory Conditions in Air Aided by a Schlenk Line

The optimized CTM reaction was further evaluated under standard laboratory conditions, i.e., without the use of a glovebox. To make CTM widely adopted and to prove the value of the methodology, a reaction of the **M3** monomer was performed at the benchtop with air-free techniques yielding **3** (see Example 3) with equal structural and speciation characteristics relative to that made in the glovebox,^27^ and with same quality of the isolated bulk materials (in terms of exclusively macrocyclic species obtained, as evidenced by both analytical GPC and HR-MALDI-TOF MS), albeit with slightly lower yield (81% vs quantitative with the use of a glovebox). Figure 7 illustrates the procedure with adequate modifications to run the CTM with a vacuum manifold.

## 4 Expected Results

### 4.1 APC Synthesis under Inert Atmosphere Inside a Glovebox

Example 1: APC with long alkyl exocyclic moiety
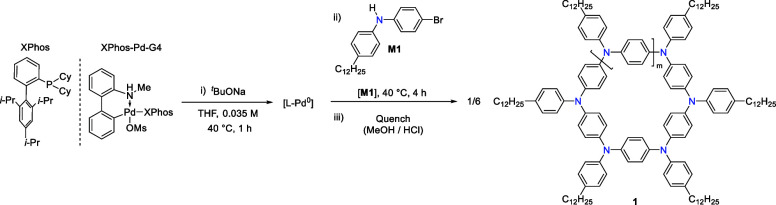


A tared 20 mL EPA vial was loaded with XPhos-Pd-G4 (8.2 mg, 0.01 mmol, 4 mol %), XPhos (4.6 mg, 0.01 mmol, 4 mol %), *t*BuONa (47.3 mg, 0.49 mmol, 2.05 equiv), and a magnetic stirrer bar. Degassed and anhydrous THF was injected into the vial via a syringe (0.035 M, e.g., *V*_T_ = 6.8 mL, Figure 2a–d; note the clear orange color). The vial was closed with a PTFE/silicone septum screw-cap and stirred at 40 °C (preheated heating block) for 1 h (Figure 2e; note the clear pale-yellow color indicating the formation of the active catalyst). Subsequently, the vial was uncapped, and **M1** (100 mg, 0.24 mmol. 1 equiv), previously weighed on a weighing boat (Figure 3a–b), was added into the catalyst solution at once (Figure 3c–d). The vial was capped again and placed into the heating block for stirring at 40 °C for 4 h (Figure 3e–h). The vial was removed from the glovebox (Figure 4a; note the reaction mixture appearance of a turbid pale-yellow color dispersion), and a solution of MeOH/HCl (1 N), 1:1 v/v (7 mL) was injected into the reaction mixture to precipitate the crude product (Figure 4b–c; note the visible beige color suspension). The APC bulk material was collected as described in Step 3 (Figure 5a, after washing/centrifugation/decantation cycles) to yield 78 mg (97%, average of two runs) of **1** as a beige powder.

After APC bulk material separation via preparative recycling GPC, the following individual nN sizes were obtained from 64 mg injected (Figure 8): **1**_**5N**_: 6.4 mg (15.0%). HRMS (MALDI-timsTOF; matrix DCTB): *m*/*z* cal. for C_120_H_165_N_5_ [M]^+^ 1676.3060, found 1676.3059. **1**_**6N**_: 17.2 mg (26.9%); ^1^H NMR (600 MHz, *d*_8_-THF) δ 7.03 (d, *J* = 8.5 Hz, 12H), 6.99 (d, *J* = 8.5 Hz, 12H), 6.91 (s, 24H), 2.53 (t, *J* = 7.8 Hz, 12H), 1.59 (p, *J* = 7.4 Hz, 12H), 1.38–1.25 (m, 108H), 0.88 (t, 18H). ^13^C{^1^H} NMR (151 MHz, *d*_8_-THF) δ 146.88, 144.20, 137.78, 130.00, 125.61, 124.60, 36.37, 33.04, 32.83, 30.80, 30.80, 30.76, 30.74, 30.68, 30.51, 30.47, 23.73, 14.61. HRMS (MALDI-timsTOF; matrix DCTB): *m*/*z* cal. for C_144_H_198_N_6_ [M]^+^ 2011.5673, found 2011.5677. **1**_**7N**_: 4.8 mg (11.3%); HRMS (MALDI-timsTOF; matrix DCTB): *m*/*z* cal. for C_168_H_231_N_7_ [M]^+^ 2346.8286, found 2346.8278. **1**_**8N**_: 2.5 mg (5.9%); HRMS (MALDI-timsTOF; matrix DCTB): *m*/*z* cal. for C_192_H_264_N_8_ [M]^+^ 2682.0899, found 2682.0899. **1**_**9N**_: 1.5 mg (3.5%); HRMS (MALDI-timsTOF; matrix DCTB): *m*/*z* cal. for C_216_H_297_N_9_ [M]^+^ 3017.3512, found 3017.3503

Example 2: APC with an electron-deficient endocyclic moiety
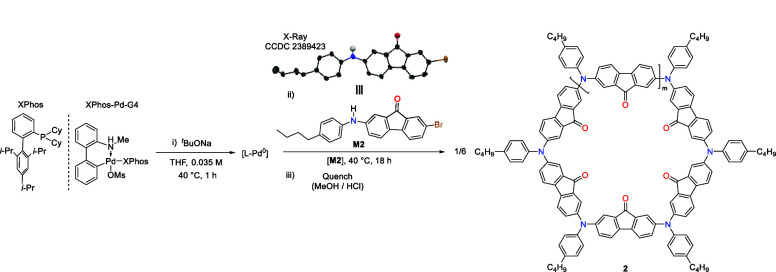


A tared 40 mL EPA vial was loaded with XPhos-Pd-G4 (12.7 mg, 0.015 mmol, 4 mol %), XPhos (7.0 mg, 0.015 mmol, 4 mol %), *t*BuONa (72.7 mg, 0.757 mmol, 2.05 equiv), and a magnetic stirrer bar. Degassed and anhydrous THF was injected into the vial via a syringe (0.035 M, e.g., V_T_ = 10.5 mL). The vial was closed with a PTFE/silicone septum screw-cap and stirred at 40 °C (preheated heating block) for 1 h. Subsequently, the vial was uncapped, and **M2** (150 mg, 0.369 mmol, 1 equiv), previously weighed on a weighing boat, was added into the catalyst solution at once. The vial was capped again and placed into the heating block for stirring at 40 °C for 18 h. The vial was removed from the glovebox, and a solution of MeOH/HCl (1 N), 1:1 v/v (11 mL) was injected into the reaction mixture to precipitate the crude product. Collection of the APC bulk material was performed as described in Step 3 to yield 110 mg (91%) of **2** as a purple powder.

After APC bulk material separation via preparative recycling GPC (Figure S7), the following individual nN sizes were obtained from 100 mg injected (Figure 9): **2**_**5N**_: 14 mg (14%); ^1^H NMR (600 MHz, *d*_8_-THF) δ 7.45 (d, *J* = 8.1 Hz, 10H), 7.29 (d, *J* = 2.3 Hz, 10H), 7.18–7.11 (m, 20H), 7.05 (d, *J* = 8.6 Hz, 10H), 2.60 (t, *J* = 7.8 Hz, 10H), 1.66–1.58 (m, 10H), 1.44–1.35 (m, 10H), 0.95 (t, *J* = 7.4 Hz, 15H). ^13^C{^1^H} NMR (151 MHz, *d*_8_-THF) δ 192.35, 148.93, 145.72, 139.95, 139.69, 137.25, 130.59, 125.56, 122.03, 120.10, 36.08, 34.89, 23.49, 14.48. HRMS (MALDI-timsTOF; matrix DCTB): *m*/*z* cal. for C_115_H_95_N_5_O_5_ [M]^+^ 1625.7328, found 1625.7320. **2**_**6N**_: 14 mg (14%); ^1^H NMR (600 MHz, *d*_8_-THF) δ 7.44 (d, *J* = 8.0 Hz, 12H), 7.24 (d, *J* = 2.4 Hz, 12H), 7.16 (d, *J* = 8.6 Hz, 12H), 7.13 (dd, *J* = 8.1, 2.2 Hz, 12H), 7.06 (d, *J* = 8.5 Hz, 12H), 2.60 (t, *J* = 7.8 Hz, 12H), 1.66–1.58 (m, 12H), 1.39 (h, *J* = 7.4 Hz, 12H), 0.95 (t, *J* = 7.3 Hz, 18H). ^13^C{^1^H} NMR (151 MHz, *d*_8_-THF) δ 192.42, 149.18, 145.53, 139.98, 139.70, 137.15, 130.61, 130.10, 125.99, 121.98, 120.09, 36.10, 34.85, 23.52, 14.48. HRMS (MALDI-timsTOF; matrix DCTB): *m*/*z* cal. for C_138_H_114_N_6_O_6_ [M]^+^ 1950.8794, found 1950.8765. **2**_**7N**_: 11 mg (11%). ^1^H NMR (700 MHz, *d*_8_-THF) δ 7.45 (d, *J* = 8.1 Hz, 14H), 7.23 (d, *J* = 2.3 Hz, 14H), 7.16 (m, 28H), 7.06 (d, *J* = 8.5 Hz, 14H), 2.61 (t, *J* = 7.8 Hz, 14H), 1.65–1.60 (m, 14H), 1.40 (h, *J* = 7.4 Hz, 14H), 0.95 (t, *J* = 7.4 Hz, 21H). HRMS (MALDI-timsTOF; matrix DCTB): *m*/*z* cal. for C_161_H_133_N_7_O_7_ [M]^+^ 2276.0261, found 2276.0231. **2**_**8N**_: 10 mg (10%); HRMS (MALDI-timsTOF; matrix DCTB): *m*/*z* cal. for C_184_H_152_N_8_O_8_ [M]^+^ 2601.1728, found 2601.1685. **2**_**9N**_: 11 mg (11%); HRMS (MALDI-timsTOF; matrix DCTB): *m*/*z* cal. for C_207_H_171_N_9_O_9_ [M]^+^ 2926.3194, found 2926.3161.

### 4.2 APC Synthesis under Standard Laboratory Conditions in Air Aided by a Schlenk Line

Example 3
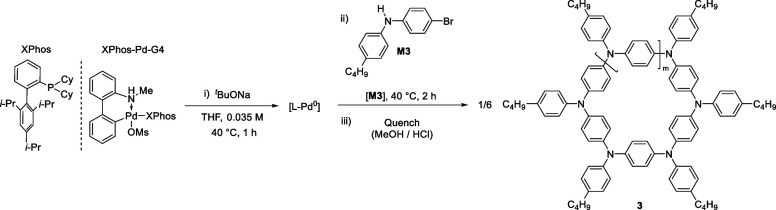


A portion of the reagents was transferred to storage vials, removed from the glovebox, and kept in a desiccator containing anhydrous P_2_O_5_ under atmospheric conditions. A 20 mL EPA vial, dried overnight in an oven, was allowed to cool to ambient temperature inside the desiccator. The vial was tared in an analytical balance and sequentially added XPhos-Pd-G4 (6.8 mg, 0.008 mmol, 4 mol %), XPhos (3.8 mg, 0.008 mmol, 4 mol %), and *t*BuONa (39 mg, 0.4 mmol, 2.05 equiv), while minimizing air exposure during the weighing (Figure 7a). A magnetic stirrer was added, and the vial was sealed with a PTFE/silicone septum screw-cap. It was then evacuated and refilled with dry argon through a syringe needle connected to a Schlenk line, and this process was repeated four times (Figure 7b). Freshly collected THF from an SPS system (degassed and anhydrous, Figure 7c) was injected into the vial via a syringe (0.035 M, e.g., V_T_ = 5.7 mL), resulting in a clear orange solution (Figure 7d). This mixture was stirred at 40 °C in a preheated oil bath for 1 h, producing a color change to clear pale-yellow, indicating the formation of the active catalyst (Figure 7e). Next, **M3** (60.3 mg, 0.2 mmol, 1 equiv) was injected neat into the catalyst solution with a microliter syringe, causing the solution to turn a clear dark orange (Figure 7f–g), and stirring was continued at 40 °C for 2 h. The vial was removed from the oil bath to cool to room temperature (Figure 7h, note the reaction mixture appearance of a turbid pale-yellow color dispersion), and a solution of MeOH/HCl (1 N), 1:1 v/v (6 mL), was injected into the mixture to quench the reaction and precipitate the crude product (Figure 7i, note the beige color suspension). The APC bulk material was collected as described in Step 3, involving washing/centrifugation/decantation cycles, yielding 36 mg (81%) of compound **3** as a pale beige powder (compared to 98% yield when the process was performed inside a glovebox). The product characterization matched previously reported data.^27^

#### Safety

Most starting materials required to prepare secondary haloaniline monomers are primary aromatic amines (PAAs). These PAAs are toxic, considered procarcinogens, and are classified as chemical hazards. The reader is referred to a recent review for a compelling summary of the range of chemical, environmental, and occupational/nonoccupational hazards.^50^ Monomers required for APC synthesis via CTM are secondary aromatic amines (SAAs), and they should be handled similarly to PAAs with regard to their hazardous potential. All operations involving the manipulation of chemical substances must be performed with care and carried out in a well-ventilated fumehood. Personal protective equipment (PPE), e.g., lab coat, safety glasses, and appropriate gloves, should be used. Proper care must be observed when handling MeOH and MeOH solutions during the purification stage.^51^ The synthetic procedures described in this Protocol require the use of syringes and needles. Hence, appropriate precautions and training must be taken when handling these items.^52^ The use of the Schlenk line and glassware subjected to pressure changes must be carried out after adequate training. The reader is referred to appropriate resources specialized in manipulating substances under anhydrous conditions^53−56^ or contemporary multimedia tutorials.^57−61^

#### Limitations

Albeit the synthesis and purification of the APCs as bulk materials is straightforward, a possible limitation of this protocol might arise from the separation stage of the individual components, i.e., APC by nN ring sizes, as it currently relies on a specialized preparative chromatography instrument (recycling GPC). Nonetheless, a do-it-yourself custom-made apparatus using inexpensive components have been reported elsewhere^62^ and hold promise to be widely adopted for these separation purposes. The scale of isolated nN APCs will be dictated by the mass loading capacity of the recycling GPC instrument and the type and number of columns, which might constitute another limitation (e.g., quantities of materials >100 mg will require multiple separation rounds for the instrument used in this work), in contrast to the synthesis that can be seamlessly scaled up (e.g., gram scale of monomer per batch). While there might be some instances where the collective properties of the bulk materials exceed those of their components, our group is currently working on alternative separation approaches to further enhance the versatility of this protocol.

## Conclusions

In this Protocol, we describe a detailed step-by-step procedure to prepare azaparacyclophanes (APCs) via the one-step catalyst-transfer macrocyclization (CTM) method. Two synthetic strategies are showcased: one employing an argon-filled glovebox to ensure replicability due to the nature of the Buchwald–Hartwig cross-coupling reaction conditions requirements (Examples 1–2), and an alternative one carried out at the benchtop under standard laboratory conditions in air (Example 3), in order to make the CTM method widely adopted by the materials science community and adjacent fields. The latter strategy displays no differences in terms of structural and speciation characteristics relative to the former strategy, with isolated yields diminished by ∼20% as the only expected disparity. Purification of isolated APC bulk materials and further separation into their individual nN ring sizes are also thoroughly described.

These oligomeric triarylamine macrocycles serve to formulate relations between their repeat unit length and physical properties, and hence, serve as models for larger and polydisperse materials.^63^ We anticipate that the synthetic possibilities opened with this protocol to make vast and functionally diverse APCs will promptly exploit their full potential and find exciting applications in next-generation organic electronics and supramolecular chemistry.

## Experimental Section

Compound 4-bromo-*N*-(4-butylphenyl)aniline (**M3**) was prepared as described previously.^27^ All other compounds were obtained from commercial sources, as noted in Table 1 and used as received unless otherwise noted (e.g., Step 1). Further experimental and instrumental details are described in the Supporting Information.

### 4-Bromo-*N*-(4-dodecylphenyl)aniline (M1)

Inside a glovebox, a Schlenk flask was charged with 4-dodecylaniline (2615 mg, 10 mmol), 4-bromophenylboronic acid (4017 mg, 20 mmol), Cu(OAc)_2_ (1907 mg, 10.5 mmol), Et_3_N (3036 mg, 30 mmol), and (*t*BuO)_2_ (2925 mg, 20 mmol) and dissolved in CH_2_Cl_2_ (50 mL). The mixture was stirred at room temperature for 24 h. The crude reaction mixture was diluted with CH_2_Cl_2_ and then filtered through a short plug of Celite. The filtrate volume was reduced to ca. 20% via rotary evaporation and then transferred into a separating funnel, washed with saturated NH_4_Cl (aq) (3×) and brine. The crude solution was dried with Na_2_SO_4_, filtered, and the volatiles evaporated under vacuo. The crude product was purified via flash column chromatography (silica gel, 100% heptane) to yield 2500 mg (60%) of **M1** as a beige solid. ^1^H NMR (700 MHz, CDCl_3_) δ 7.34–7.29 (m, 2H), 7.10 (d, *J* = 8.5 Hz, 2H), 6.99 (d, *J* = 8.5 Hz, 2H), 6.91–6.86 (m, 2H), 5.67 (s, 1H), 2.55 (t, *J* = 7.7 Hz, 2H), 1.62–1.56 (m, 2H), 1.36–1.23 (m, 18H), 0.88 (t, *J* = 7.1 Hz, 3H). ^13^C{^1^H} NMR (176 MHz, CDCl_3_) δ 143.28, 139.89, 137.07, 132.25, 129.46, 119.39, 118.39, 112.07, 35.42, 32.08, 31.79, 29.83, 29.82 29.80, 29.77, 29.68, 29.51, 29.46, 22.85, 14.27. HRMS (ESI): *m*/*z* calc. for C_24_H_35_NBr [M + H]^+^ 416.1947, found 416.1949

### 2-Bromo-7-((4-butylphenyl)amino)-9*H*-fluoren-9-one (M2)

Inside a glovebox, a Schlenk flask was charged with 4-butylaniline (426 mg, 2.85 mmol), 2-bromo-7-iodo-9*H*-fluoren-9-one (1099 mg, 2.85 mmol), sodium trimethylsilanolate (656.4 mg, 2.05 mmol), Pd_2_dba_3_ (32.7 mg, 1.25 mol %), and XantPhos (41.3 mg, 2.5 mol %) and dissolved in toluene (22 mL). The mixture was stirred at 75 °C for 24 h. The crude reaction mixture was diluted with toluene and then filtered through a short plug of Celite. The filtrate solution was evaporated via rotary evaporation, dissolved in CH_2_Cl_2_ and then transferred into a separating funnel, and washed with water (3×) and brine. The crude solution was dried with Na_2_SO_4_, filtered and the volatiles evaporated under vacuo. The crude product was purified via flash column chromatography (silica gel, 75% CH_2_Cl_2_/heptane) to yield 1000 mg (86%) of **M2** as a dark red fluffy solid. Single crystals suitable for X-ray crystallography were obtained from the slow diffusion of methanol into a solution of the product in CH_2_Cl_2_ at ambient temperature. ^1^H NMR (600 MHz, *d*_8_-THF) δ 7.66 (s, 1H), 7.61 (d, *J* = 1.9 Hz, 1H), 7.57 (dd, *J* = 7.9, 1.9 Hz, 1H), 7.42 (d, *J* = 8.1 Hz, 1H), 7.38 (d, *J* = 7.9 Hz, 1H), 7.23 (d, *J* = 2.3 Hz, 1H), 7.13–7.07 (m, 3H), 7.06–7.01 (m, 2H), 2.57 (t, *J* = 7.7 Hz, 2H), 1.63–1.55 (m, 2H), 1.37 (h, *J* = 7.4 Hz, 2H), 0.94 (t, *J* = 7.4 Hz, 3H). ^13^C{^1^H} NMR (151 MHz, *d*_8_-THF) δ 192.53, 147.91, 145.47, 140.99, 138.02, 137.39, 137.05, 136.52, 134.63, 130.14, 127.69, 122.69, 121.81, 121.44, 120.98, 120.69, 112.03, 35.99, 35.01, 23.36, 14.48. HRMS (ESI): *m*/*z* calc. for C_23_H_20_NOBr [M + H]^+^ 406.0801, found 406.0795

## Supporting Information Available

Additional experimental and instrumental details, NMR spectra for all compounds, X-ray crystallography (PDF)

## References

[ref1] Li P.; Jia Y.; Chen P. Design and Synthesis of New Type of Macrocyclic Architectures Used for Optoelectronic Materials and Supramolecular Chemistry. Chem.—Eur. J. 2023, 29 (54), e202300300 10.1002/chem.202300300. 37439485

[ref2] Shi Q.; Wang X.; Liu B.; Qiao P.; Li J.; Wang L. Macrocyclic host molecules with aromatic building blocks: the state of the art and progress. Chem. Commun. 2021, 57 (93), 12379–12405. 10.1039/D1CC04400A. 34726202

[ref3] Talukdar D.; Kumar J. M.; Gole B. Self-assembled Macrocycles: Design Strategies and Emerging Functions. Cryst. Growth Des. 2023, 23 (11), 7582–7611. 10.1021/acs.cgd.3c00677.

[ref4] Zhu H.; Chen L.; Sun B.; Wang M.; Li H.; Stoddart J. F.; Huang F. Applications of macrocycle-based solid-state host–guest chemistry. Nat. Rev. Chem. 2023, 7 (11), 768–782. 10.1038/s41570-023-00531-9. 37783822

[ref5] Ball M.; Zhang B.; Zhong Y.; Fowler B.; Xiao S.; Ng F.; Steigerwald M.; Nuckolls C. Conjugated Macrocycles in Organic Electronics. Acc. Chem. Res. 2019, 52 (4), 1068–1078. 10.1021/acs.accounts.9b00017. 30869865

[ref6] Iyoda M.; Yamakawa J.; Rahman M. J. Conjugated Macrocycles: Concepts and Applications. Angew. Chem., Int. Ed. 2011, 50 (45), 10522–10553. 10.1002/anie.201006198. 21960431

[ref7] Liu Z.; Nalluri S. K. M.; Stoddart J. F. Surveying macrocyclic chemistry: from flexible crown ethers to rigid cyclophanes. Chem. Soc. Rev. 2017, 46 (9), 2459–2478. 10.1039/C7CS00185A. 28462968

[ref8] Roy I.; David A. H. G.; Das P. J.; Pe D. J.; Stoddart J. F. Fluorescent cyclophanes and their applications. Chem. Soc. Rev. 2022, 51 (13), 5557–5605. 10.1039/D0CS00352B. 35704949

[ref9] Wu G.; Yang Y.-W. Macrocycle-based fluorochromic systems. Cell Rep. Phys. Sci. 2024, 5 (3), 101873 10.1016/j.xcrp.2024.101873.

[ref10] Höger S. Highly efficient methods for the preparation of shape-persistent macrocyclics. J. Polym. Sci., Part A: Polym. Chem. 1999, 37 (15), 2685–2698. 10.1002/(SICI)1099-0518(19990801)37:15<2685::AID-POLA1>3.0.CO;2-S.

[ref11] Ito A. Macrocyclic oligoarylamines as hole- and spin-containing scaffolds for molecule-based electronics. J. Mater. Chem. C 2016, 4 (21), 4614–4625. 10.1039/C6TC00973E.

[ref12] Takemura H. [1_n_]Paracyclophanes. Curr. Org. Chem. 2009, 13 (16), 1633–1653. 10.2174/138527209789578117.

[ref13] Shahnawaz S.; Sudheendran Swayamprabha S.; Nagar M. R.; Yadav R. A. K.; Gull S.; Dubey D. K.; Jou J.-H. Hole-transporting materials for organic light-emitting diodes: an overview. J. Mater. Chem. C 2019, 7 (24), 7144–7158. 10.1039/C9TC01712G.

[ref14] Shirota Y. Organic materials for electronic and optoelectronic devices. J. Mater. Chem. 2000, 10 (1), 1–25. 10.1039/a908130e.

[ref15] Shirota Y. Photo- and electroactive amorphous molecular materials—molecular design, syntheses, reactions, properties, and applications. J. Mater. Chem. 2005, 15 (1), 75–93. 10.1039/B413819H.

[ref16] Calió L.; Kazim S.; Grätzel M.; Ahmad S. Hole-Transport Materials for Perovskite Solar Cells. Angew. Chem., Int. Ed. 2016, 55 (47), 14522–14545. 10.1002/anie.201601757. 27739653

[ref17] Ning Z.; Tian H. Triarylamine: a promising core unit for efficient photovoltaic materials. Chem. Commun. 2009, 37, 5483–5495. 10.1039/b908802d. 19753339

[ref18] Rombach F. M.; Haque S. A.; Macdonald T. J. Lessons learned from spiro-OMeTAD and PTAA in perovskite solar cells. Energy Environ. Sci. 2021, 14 (10), 5161–5190. 10.1039/D1EE02095A.

[ref19] Wang J.; Liu K.; Ma L.; Zhan X. Triarylamine: Versatile Platform for Organic, Dye-Sensitized, and Perovskite Solar Cells. Chem. Rev. 2016, 116 (23), 14675–14725. 10.1021/acs.chemrev.6b00432. 27960267

[ref20] Pron A.; Gawrys P.; Zagorska M.; Djurado D.; Demadrille R. Electroactive materials for organic electronics: preparation strategies, structural aspects and characterization techniques. Chem. Soc. Rev. 2010, 39 (7), 2577–2632. 10.1039/b907999h. 20393644

[ref21] Song Y.; Di C.-a.; Xu W.; Liu Y.; Zhang D.; Zhu D. New semiconductors based on triphenylamine with macrocyclic architecture: synthesis, properties and applications in OFETs. J. Mater. Chem. 2007, 17 (42), 4483–4491. 10.1039/b708887f.

[ref22] Wang C.; Dong H.; Hu W.; Liu Y.; Zhu D. Semiconducting π-Conjugated Systems in Field-Effect Transistors: A Material Odyssey of Organic Electronics. Chem. Rev. 2012, 112 (4), 2208–2267. 10.1021/cr100380z. 22111507

[ref23] Gu C.; Jia A.-B.; Zhang Y.-M.; Zhang S. X.-A. Emerging Electrochromic Materials and Devices for Future Displays. Chem. Rev. 2022, 122 (18), 14679–14721. 10.1021/acs.chemrev.1c01055. 35980039 PMC9523732

[ref24] Yen H.-J.; Liou G.-S. Recent advances in triphenylamine-based electrochromic derivatives and polymers. Polym. Chem. 2018, 9 (22), 3001–3018. 10.1039/C8PY00367J.

[ref25] Yen H.-J.; Liou G.-S. Design and preparation of triphenylamine-based polymeric materials towards emergent optoelectronic applications. Prog. Polym. Sci. 2019, 89, 250–287. 10.1016/j.progpolymsci.2018.12.001.

[ref26] Mao L.; Zhou M.; Shi X.; Yang H.-B. Triphenylamine (TPA) radical cations and related macrocycles. Chin. Chem. Lett. 2021, 32 (11), 3331–3341. 10.1016/j.cclet.2021.05.004.

[ref27] Ayuso-Carrillo J.; Fina F.; Galleposo E. C.; Ferreira R. R.; Mondal P. K.; Ward B. D.; Bonifazi D. One-Step Catalyst-Transfer Macrocyclization: Expanding the Chemical Space of Azaparacyclophanes. J. Am. Chem. Soc. 2024, 146 (24), 16440–16457. 10.1021/jacs.4c02319. 38848549 PMC11191698

[ref28] Ito A.; Yokoyama Y.; Aihara R.; Fukui K.; Eguchi S.; Shizu K.; Sato T.; Tanaka K. Preparation and Characterization of *N*-Anisyl-Substituted Hexaaza[1_6_]paracyclophane. Angew. Chem., Int. Ed. 2010, 49 (44), 8205–8208. 10.1002/anie.201002165. 20859974

[ref29] Fang Z.; Samoc M.; Webster R. D.; Samoc A.; Lai Y.-H. Triphenylamine derivatized phenylacetylene macrocycle with large two-photon absorption cross-section. Tetrahedron Lett. 2012, 53 (36), 4885–4888. 10.1016/j.tetlet.2012.07.003.

[ref30] Kim K.; Ohata R.; Kanehashi S.; Tsuchiya K.; Ogino K. Hole Transporting Properties of Cyclic Pentamer of 4-Butyltriphenylamine. Chem. Lett. 2017, 46 (8), 1145–1147. 10.1246/cl.170412.

[ref31] Kurata R.; Sakamaki D.; Uebe M.; Kinoshita M.; Iwanaga T.; Matsumoto T.; Ito A. Isolable Triradical Trication of Hexaaza[1_6_]paracyclophane with Embedded 9,10-Anthrylenes: A Frustrated Three-Spin System. Org. Lett. 2017, 19 (16), 4371–4374. 10.1021/acs.orglett.7b02088. 28783366

[ref32] Louie S.; Zhong Y.; Bao S. T.; Schaack C.; Montoya A.; Jin Z.; Orchanian N. M.; Liu Y.; Lei W.; Harrison K.; Hone J.; Angerhofer A.; Evans A. M.; Nuckolls C. P. Coaxially Conductive Organic Wires Through Self-Assembly. J. Am. Chem. Soc. 2023, 145 (9), 4940–4945. 10.1021/jacs.2c12437. 36852948

[ref33] Picini F.; Schneider S.; Gavat O.; Vargas Jentzsch A.; Tan J.; Maaloum M.; Strub J.-M.; Tokunaga S.; Lehn J.-M.; Moulin E.; Giuseppone N. Supramolecular Polymerization of Triarylamine-Based Macrocycles into Electroactive Nanotubes. J. Am. Chem. Soc. 2021, 143 (17), 6498–6504. 10.1021/jacs.1c00623. 33834779

[ref34] Tsuchiya K.; Miyaishi H.; Ogino K. Facile Preparation of Macrocycles with Triphenylamine Backbone via C–N Coupling Reaction. Chem. Lett. 2011, 40 (9), 931–933. 10.1246/cl.2011.931.

[ref35] Yang T.-F.; Chiu K. Y.; Cheng H.-C.; Lee Y. W.; Kuo M. Y.; Su Y. O. Studies on the Structure of *N*-Phenyl-Substituted Hexaaza[1_6_]paracyclophane: Synthesis, Electrochemical Properties, And Theoretical Calculation. J. Org. Chem. 2012, 77 (19), 8627–8633. 10.1021/jo301436g. 22950953

[ref36] Bruno N. C.; Niljianskul N.; Buchwald S. L. N-Substituted 2-Aminobiphenylpalladium Methanesulfonate Precatalysts and Their Use in C–C and C–N Cross-Couplings. J. Org. Chem. 2014, 79 (9), 4161–4166. 10.1021/jo500355k. 24724692 PMC4017611

[ref37] Altman R. A.; Fors B. P.; Buchwald S. L. Pd-catalyzed amination reactions of aryl halides using bulky biarylmonophosphine ligands. Nat. Protoc. 2007, 2 (11), 2881–2887. 10.1038/nprot.2007.414. 18007623

[ref38] Lo C. K.; Wolfe R. M. W.; Reynolds J. R. From Monomer to Conjugated Polymer: A Perspective on Best Practices for Synthesis. Chem. Mater. 2021, 33 (13), 4842–4852. 10.1021/acs.chemmater.1c01142.

[ref39] Firsan S. J.; Sivakumar V.; Colacot T. J. Emerging Trends in Cross-Coupling: Twelve-Electron-Based L_1_Pd(0) Catalysts, Their Mechanism of Action, and Selected Applications. Chem. Rev. 2022, 122 (23), 16983–17027. 10.1021/acs.chemrev.2c00204. 36190916 PMC9756297

[ref40] Ingoglia B. T.; Wagen C. C.; Buchwald S. L. Biaryl monophosphine ligands in palladium-catalyzed C–N coupling: An updated User’s guide. Tetrahedron 2019, 75 (32), 4199–4211. 10.1016/j.tet.2019.05.003. 31896889 PMC6939672

[ref41] Surry D. S.; Buchwald S. L. Biaryl Phosphane Ligands in Palladium-Catalyzed Amination. Angew. Chem., Int. Ed. 2008, 47 (34), 6338–6361. 10.1002/anie.200800497. PMC3517088 18663711

[ref42] Surry D. S.; Buchwald S. L. Dialkylbiaryl phosphines in Pd-catalyzed amination: a user’s guide. Chem. Sci. 2011, 2 (1), 27–50. 10.1039/C0SC00331J. 22432049 PMC3306613

[ref43] Barder T. E.; Buchwald S. L. Rationale Behind the Resistance of Dialkylbiaryl Phosphines toward Oxidation by Molecular Oxygen. J. Am. Chem. Soc. 2007, 129 (16), 5096–5101. 10.1021/ja0683180. 17388595

[ref44] Pangborn A. B.; Giardello M. A.; Grubbs R. H.; Rosen R. K.; Timmers F. J. Safe and Convenient Procedure for Solvent Purification. Organometallics 1996, 15 (5), 1518–1520. 10.1021/om9503712.

[ref45] Inoue R.; Yamaguchi M.; Murakami Y.; Okano K.; Mori A. Revisiting of Benzophenone Ketyl Still: Use of a Sodium Dispersion for the Preparation of Anhydrous Solvents. ACS Omega 2018, 3 (10), 12703–12706. 10.1021/acsomega.8b01707. 30411016 PMC6210062

[ref46] Nichols L. Transferring Methods - Inert Atmospheric Methods. LibreTexts https://chem.libretexts.org/@go/page/93193 (accessed 16-08-2024).

[ref47] Armarego W. L. F. Purification of laboratory chemicals, 8th ed.; Butterworth-Heinemann: Oxford, 2017.

[ref48] Hartwig J. F.; Shaughnessy K. H.; Shekhar S.; Green R. A. Palladium-catalyzed amination of aryl halides. In Organic Reactions; Denmark S. E., Ed. John Wiley & Sons, Inc.: 2020; pp 853–958.

[ref49] Ruiz-Castillo P.; Buchwald S. L. Applications of Palladium-Catalyzed C–N Cross-Coupling Reactions. Chem. Rev. 2016, 116 (19), 12564–12649. 10.1021/acs.chemrev.6b00512. 27689804 PMC5070552

[ref50] Gheni S. A.; Ali M. M.; Ta G. C.; Harbin H. J.; Awad S. A. Toxicity, Hazards, and Safe Handling of Primary Aromatic Amines. ACS Chem. Health Saf. 2024, 31 (1), 8–21. 10.1021/acs.chas.3c00073.

[ref51] Koshiba Y.; Wakui K.; Ito M. Lessons Learned–Splashing Incidents of Methanol and ortho-Chlorobenzaldehyde into Eyes and Faces Due to Accidental Detachment of Luer Slip Syringes and Needles. ACS Chem. Health Saf. 2024, 31 (3), 222–228. 10.1021/acs.chas.4c00011.

[ref52] Chandra T.; Zebrowski J. P.; Lenertz L. Y. Safe Handling of Cannulas and Needles in Chemistry Laboratories. ACS Chem. Health Saf. 2022, 29 (2), 175–183. 10.1021/acs.chas.1c00069.

[ref53] Borys A. M. An Illustrated Guide to Schlenk Line Techniques. Organometallics 2023, 42 (3), 182–196. 10.1021/acs.organomet.2c00535.

[ref54] Millar S. Tips and Tricks for the Lab: Air-Sensitive Techniques. ChemistryViews [Online], 2013. 10.1002/chemv.201300002 (accessed 29-08-2024).

[ref55] Salzer A. Laboratory Techniques of Organometallic Chemistry. In Synthetic Methods of Organometallic and Inorganic Chemistry. Vol. 1: Literature, Laboratory Techniques, and Common Starting Materials; Herrmann W. A., Salzer A., Eds.; Georg Thieme Verlag KG: Stuttgart, 1996.

[ref56] Wayda A. L.; Darensbourg M. Y. Experimental Organometallic Chemistry. A Practicum in Synthesis and Characterization; American Chemical Society: Washington, DC, 1987; Vol. 357.

[ref57] JoVE. Schlenk Lines Transfer of Solvents. JoVE Science Education Database; MyJove Corp.: Cambridge, MA, 2023. https://app.jove.com/v/5679/schlenk-lines-transfer-of-solvents (accessed 27-08-2024).

[ref58] JoVE. Degassing Liquids with Freeze-Pump-Thaw Cycling. JoVE Science Education Database; MyJove Corp.: Cambridge, MA, 2023. https://app.jove.com/v/5639/degassing-liquids-with-freeze-pump-thaw-cycling (accessed 27-08-2024).

[ref59] JoVE. Handling Air- and Water-Sensitive Chemicals Using a Schlenk Line. JoVE Science Education Database; MyJove Corp.: Cambridge, MA, 2023. https://app.jove.com/v/10376/handling-air-and-water-sensitive-chemicals-using-a-schlenk-line (accessed 27–08–2024).

[ref60] JoVE. Preparing Anhydrous Reagents and Equipment. JoVE Science Education Database; MyJove Corp.: Cambridge, MA, 2023. https://app.jove.com/v/10227/preparing-anhydrous-reagents-and-equipment (accessed 27-08-2024).

[ref61] JoVE. Glovebox and Impurity Sensors. JoVE Science Education Database; MyJove Corp.: Cambridge, MA, 2023. https://app.jove.com/v/10317/glovebox-and-impurity-sensors (accessed 27-08-2024).

[ref62] Kleine A.; Kimmig J.; Mankel C.; Schubert U. S.; Jäger M. Low-Pressure Preparative Size-Exclusion Chromatography: from Narrow Disperse Polymer to Monodisperse Oligomer Batches. Macromolecules 2024, 57 (13), 6090–6099. 10.1021/acs.macromol.4c00448.

[ref63] Müllen K.; Wegner G. Electronic Materials: The Oligomer Approach; Wiley-VCH: Weinheim, 1998.

