# Supplementary material for: Catalyst-Transfer Macrocyclization Protocol: Synthesis of π-Conjugated Azaparacyclophanes Made Easy
Source: JACS Au. 2025 Mar 7;5(3):1482–98. doi: 10.1021/jacsau.5c00109 (PMC11937971; doi:10.1021/jacsau.5c00109)
Supplement: Supplementary file 1 — au5c00109_si_001.pdf [file au5c00109_si_001.pdf]

Supporting Information for

Catalyst-Transfer Macrocyclization Protocol: Synthesis of π-Conjugated Azaparacyclophanes Made Easy

Josue Ayuso-Carrillo, Davide Bonifazi*

Institute of Organic Chemistry, University of Vienna. Währinger Strasse 38, A-1090. Vienna, Austria

E: davide.bonifazi@univie.ac.at

Table of Contents

1. General considerations …………………………………………………………………... S2
2. NMR spectra of monomers ………………………………………………………………… S3
3. NMR spectra of Azaparacyclophanes (APCs) ……….…………………………………….. S6
4. X-Ray Crystallography ……………………………………………………………………….. S11
5. References …………………………………………………………………………………….. S12

1. General Considerations

Materials and Methods. All synthetic manipulations of air-sensitive compounds were carried out under an argon atmosphere using standard Schlenk techniques or in an argon-filled MBraun glovebox (O_2 and H_2O levels below 1.0 ppm). Glassware was dried overnight in a hot oven (120 °C) and heated under vacuum before use. Aniline derivatives were dried from CaH_2 under argon and stored in the glovebox. Toluene, CH_2Cl_2, and THF were dried using a MBraun solvent purification system, stored in Straus flasks over activated 3 Å molecular sieves, and were freeze-pump-thaw degassed prior to use. *t*BuONa was heated at 120 °C under reduced pressure (1×10^{-2} mbar) for at least 8 h and stored in the glovebox. Compound **M3** was synthesized according to literature procedures.[1] All other compounds were purchased from commercial vendors and used as received (e.g., Sigma-Aldrich, TCI, BLD Pharm, Fluorochem, Fisher Scientific, ABCR, Acros Organics, Strem). Room (ambient) temperature (RT) refers to 24 °C (± 1 °C).

Flash column chromatography was performed via a semi-automatic Biotage Isolera One flash chromatography system using silica gel (Macherey-Nagel Kieselgel 60, 0.04 – 0.063 mm).

Preparative Recycling Gel Permeation Chromatography. Purification of azaparacyclophanes mixtures were performed on a Japan Analytical Industry Co, Ltd. LaboACE LC-7080-Plus liquid chromatograph equipped with UV detectors (290 nm, 320 nm, 354 nm, 410 nm), two columns (JAIGEL-2HR and JAIGEL-2.5 HR) and precolumn JAIGEL-HR-P (ID 8 mm x 40 mm). Standard operating conditions were as follows: eluent toluene, flow rate 10 mL/min, column pressure ≤9 MPa, injection volume 5 mL, ambient temperature.

Nuclear magnetic resonance (NMR) characterizations were performed at the NMR centre of the University of Vienna. All NMR spectra were recorded on a 600 Bruker AV III (^1H, 600 MHz; ^{13}C, 150.9 MHz) or a 700 Bruker Avance Neo (^1H, 700 MHz; ^{13}C, 176.1 MHz) spectrometers. Carbon spectra were recorded with a complete decoupling for the proton. Proton and carbon chemical shifts are reported in parts per million (ppm, δ scale) according to tetramethylsilane ($\delta_H = \delta_C = 0$ ppm) using the solvent residual signal as an internal reference (e.g., $CHCl_3$: $\delta_H = 7.26$ ppm, $\delta_C = 77.16$ ppm, d_8-THF: $\delta_H = 3.58$ ppm, $\delta_C = 67.21$ ppm). All coupling constants (*J*) are reported in Hz. Multiplicity of signals are indicated as “s”, “d”, “dd”, “ddd”, “t”, “q”, “p”, “h”, “m” for singlet, doublet, doublet of doublets, doublet of doublets of doublets, triplet, quartet, pentet, heptet and multiplet, respectively. Unless otherwise stated all NMR spectra are recorded at 293 K.

Gel-Permeation Chromatography. Analytical GPC measurements were performed on an Agilent Technologies instrument equipped with RI detector and two SDV columns (Porosity 1000 and 100000 Å; Polymer Standard Services) with THF as the eluent (~1 mg mL^{-1}, flow rate 1 mL/min, 40 °C). A 10-point calibration based on polystyrene standards (Polystyrene, ReadyCal Kit, Polymer Standard Services) was applied for determination of molecular weights and dispersities. (Cirrus GPC

Offline GPC/SEC Software version 3.4.2 by Agilent Technologies) of the isolated APCs to confirm sample uniformity/purity after separation by recycling GPC.

MALDI-TOF Analysis. High-resolution MALDI-TOF MS analyses were performed using a Bruker timsTOF fleX ESI/MALDI dual source - trapped ion mobility separation - Qq-TOF mass spectrometer in positive ion mode. The sum formulas of the detected ions were determined using Bruker Compass DataAnalysis 5.3 based on the mass accuracy ($\Delta m/z \leq 5$ ppm) and isotopic pattern matching (SmartFormula algorithm). One microliter of a solution of the matrix (*trans*-2-[3-(4-*t*-butyl-phenyl)-2-methyl-2-propenylidene]malonitrile, DCTB) in THF (10 mg/mL) was spotted onto a well of the MALDI plate, and the solvent was allowed to evaporate. Sample solutions (1 mg/mL in THF) were prepared, and 1 μL of this solution was spotted onto the well by a layering method. The solvent was evaporated prior to analysis. Data were collected in positive polarity mode.

HR-MS analysis. High resolution mass spectrometry analyses were performed by the Mass Spectrometry Centre, Fakultät für Chemie, University of Vienna. ESI mass spectra (m/z 50-1900) were obtained on a Bruker maXis UHR ESI-Qq-TOF mass spectrometer in the positive ion mode by direct infusion. The sum formulas of the detected ions were determined using Bruker Compass DataAnalysis 4.1 based on the mass accuracy ($\Delta m/z \leq 5$ ppm) and isotopic pattern matching (SmartFormula algorithm).

Single Crystal X-Ray Crystallography. Single crystal X-ray structure determination was performed at the Centre for X-Ray Structure Analysis, Fakultät für Chemie, University of Vienna (**M2**).

2. NMR spectra of monomers

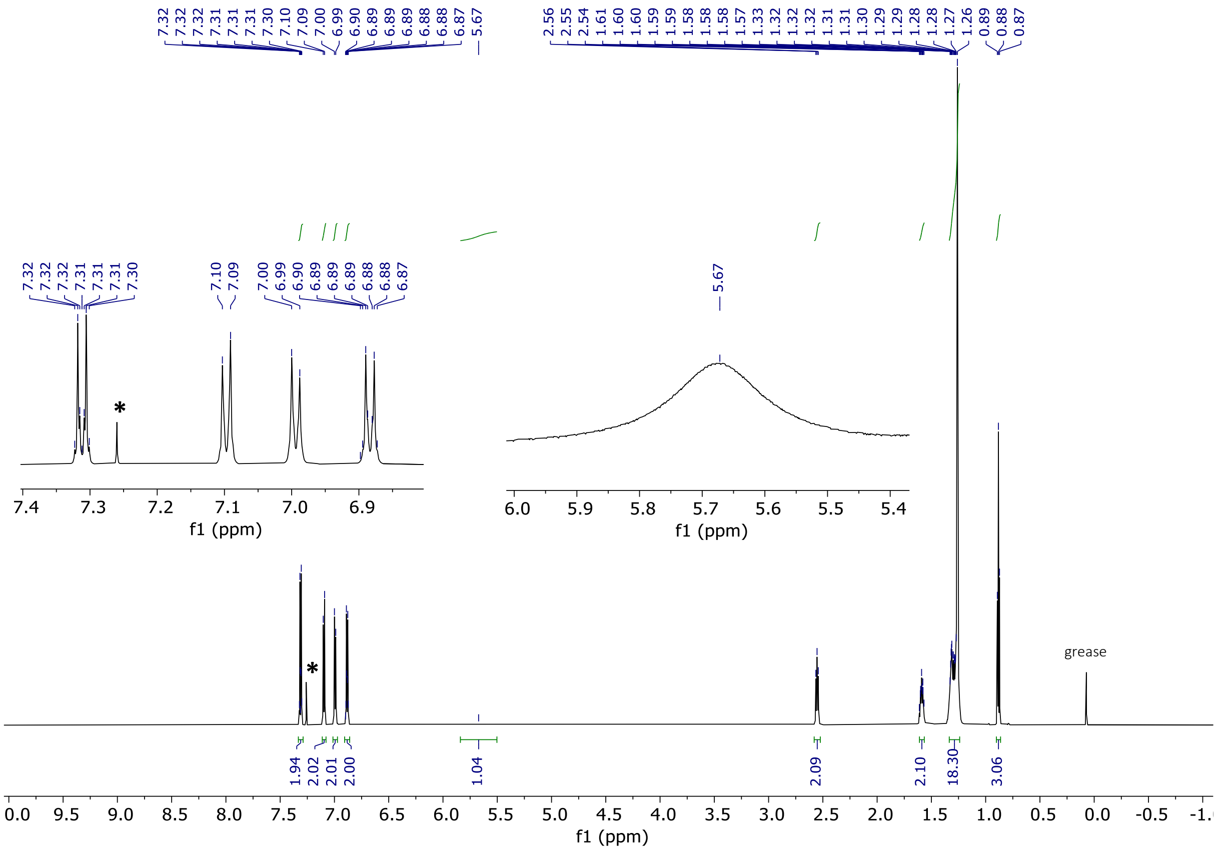

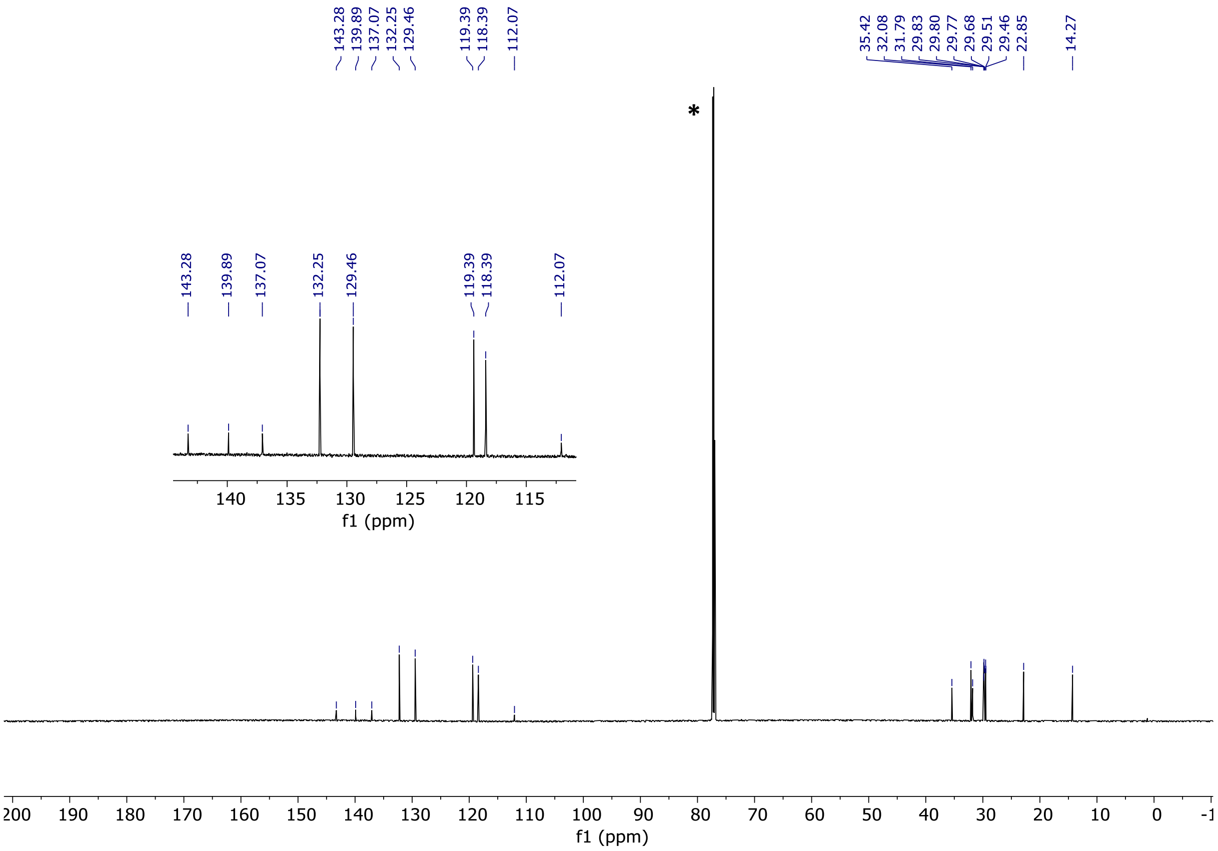

Figure S1. ^{1}H (top) and ^{13}C{^{1}H} (bottom) NMR spectra ($CDCl_3$) of **M1** (* = residual solvent).

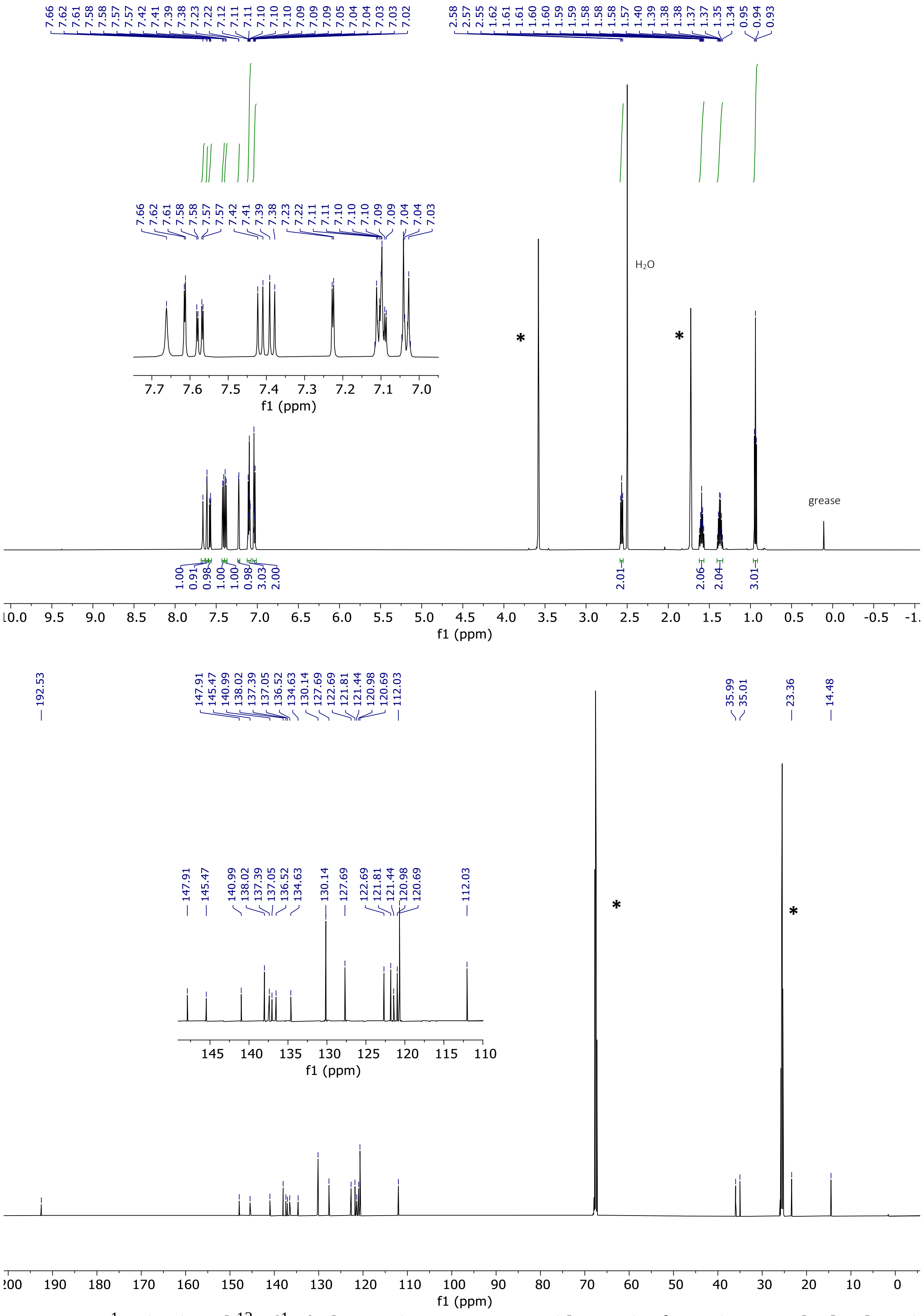

Figure S2. ^{1}H (top) and $^{13}C\{^{1}H\}$ (bottom) NMR spectra (d_8-THF) of **M2** (* = residual solvent).

3. NMR spectra of Azaparacyclophanes (APCs)

1_{6N}:

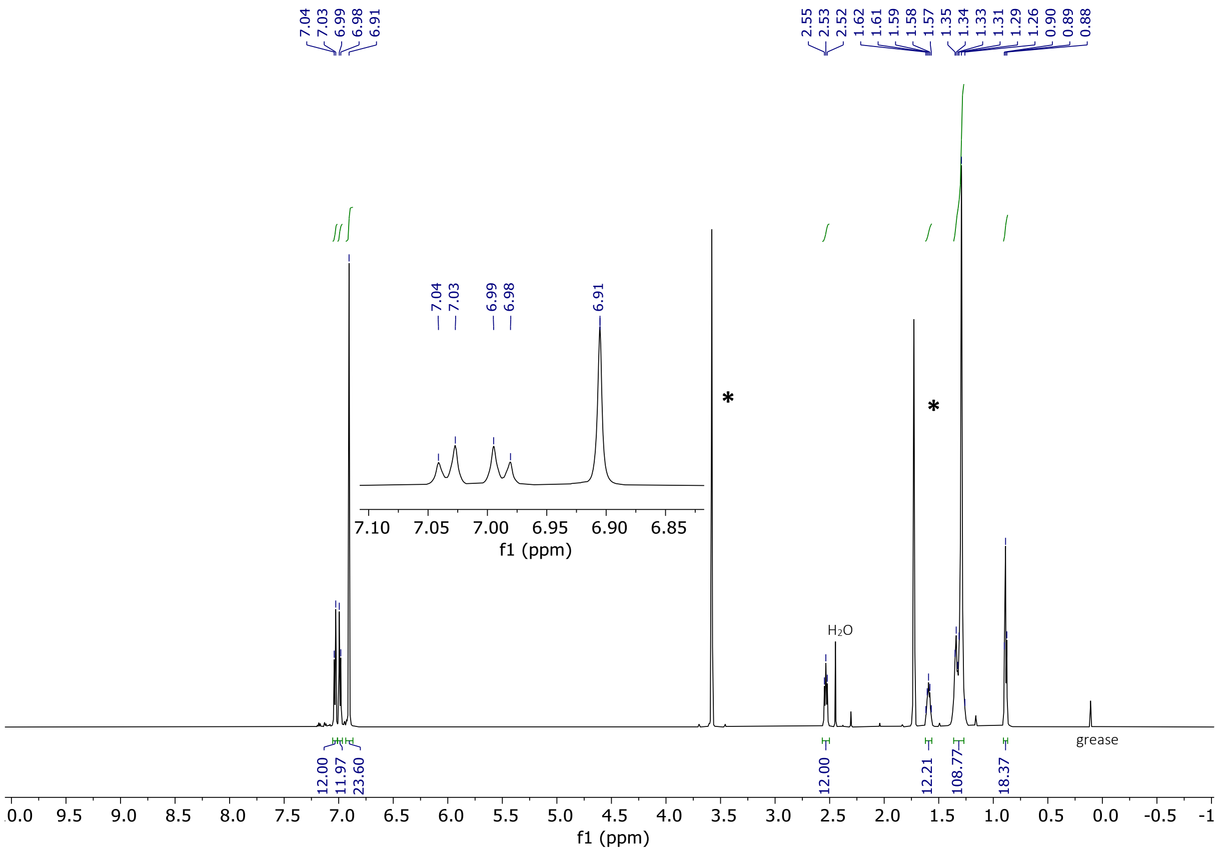

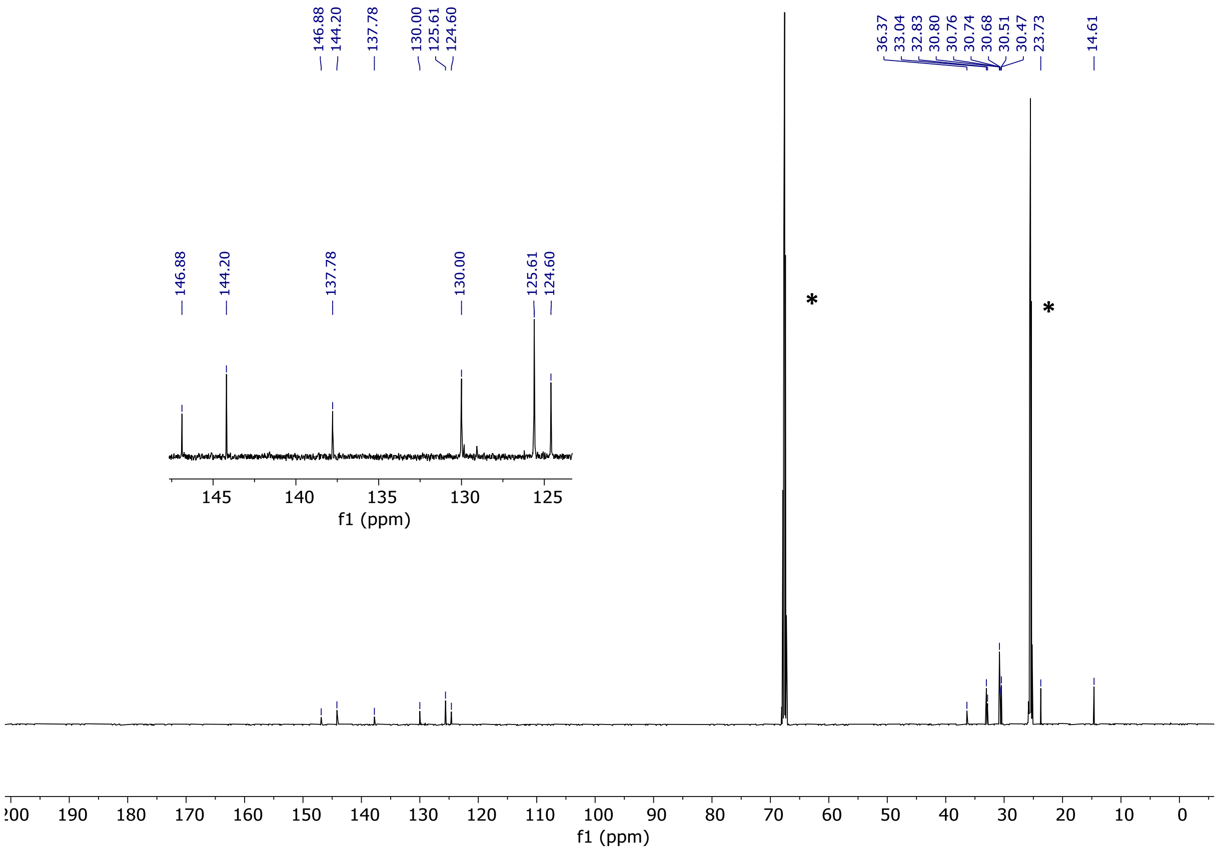

Figure S3. ^{1}H (top) and ^{13}C{^{1}H} (bottom) NMR spectra (d_8-THF) of **1_{6N}** (* = residual solvent).

2$_{5N}$:

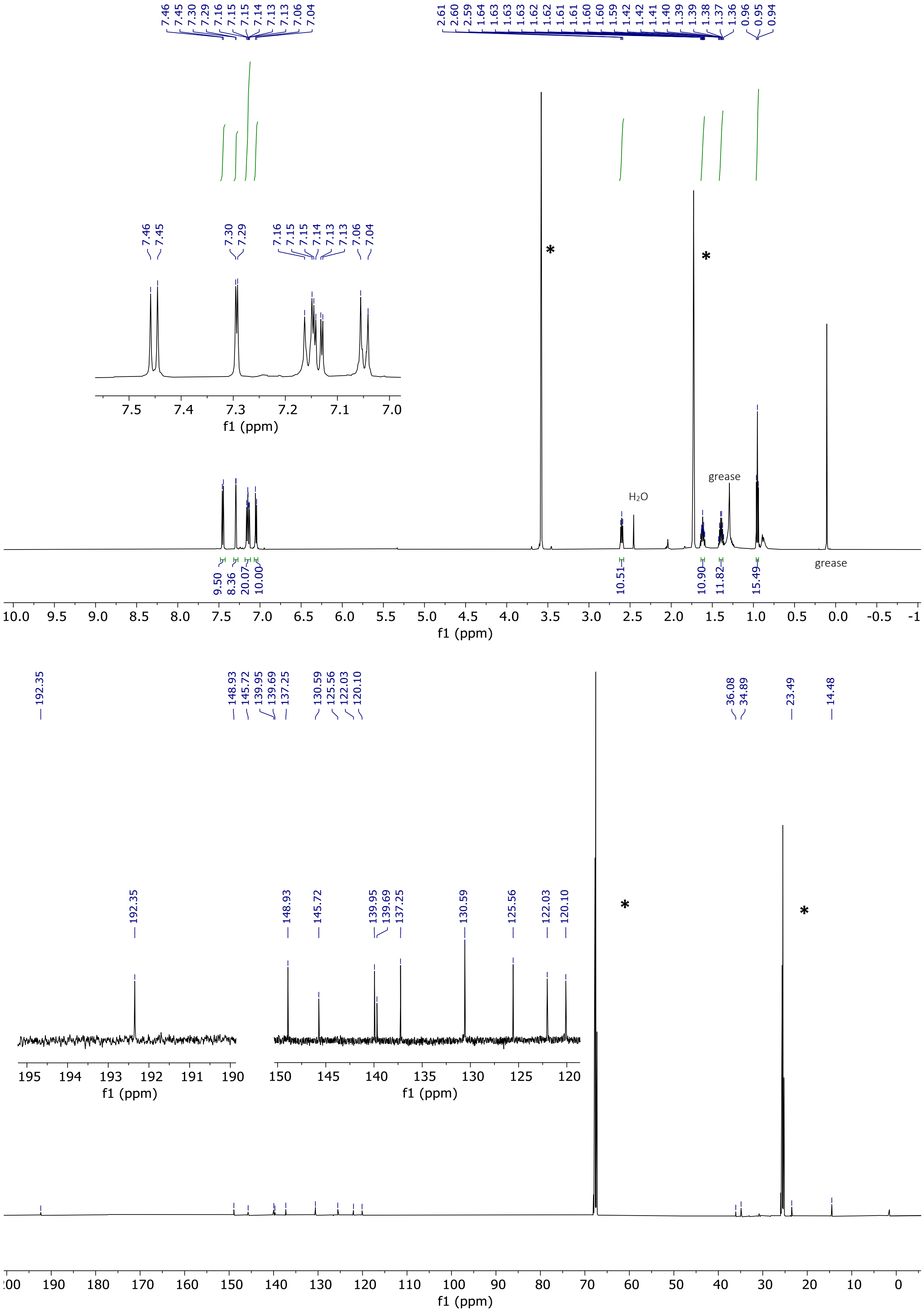

Figure S4. ^{1}H (top) and $^{13}C\{^{1}H\}$ (bottom) NMR spectra (d_8-THF) of **2$_{5N}$** (* = residual solvent).

$\mathbf{2_{6N}}$:

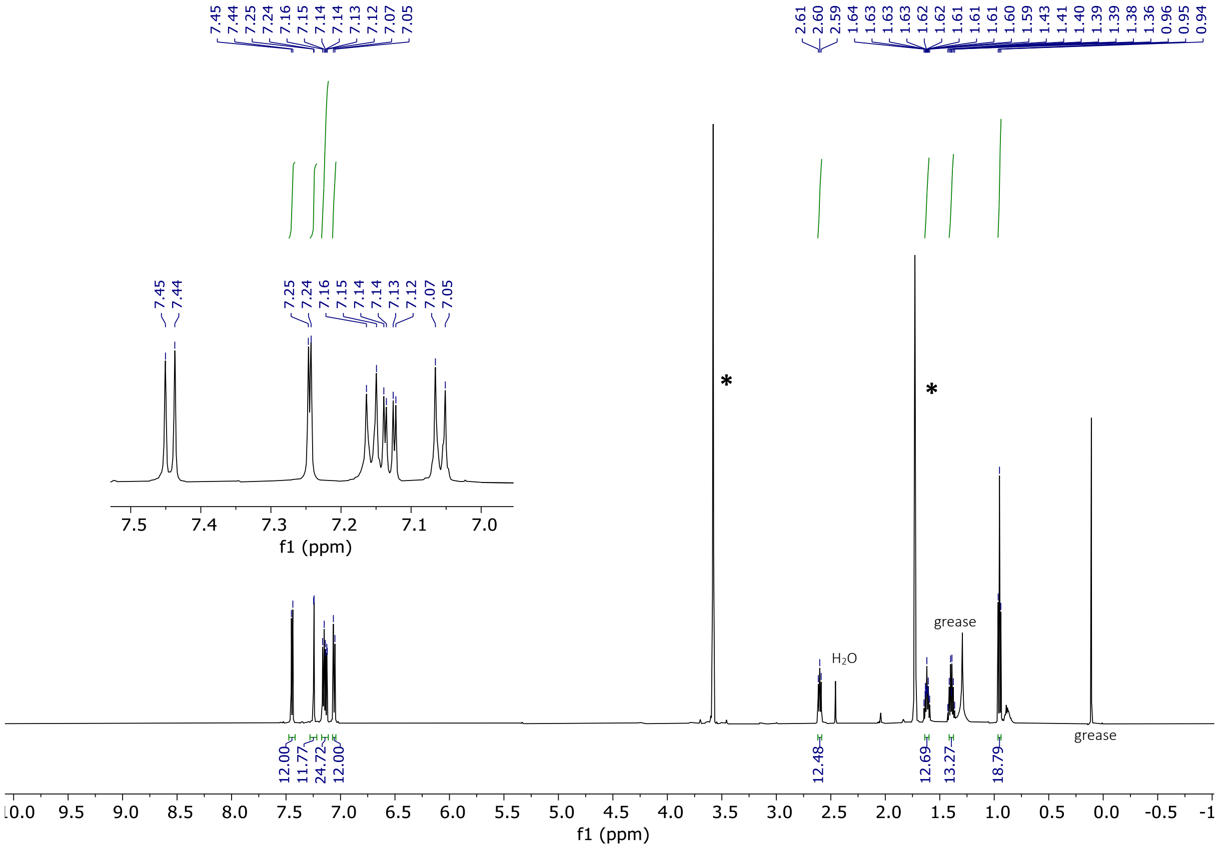

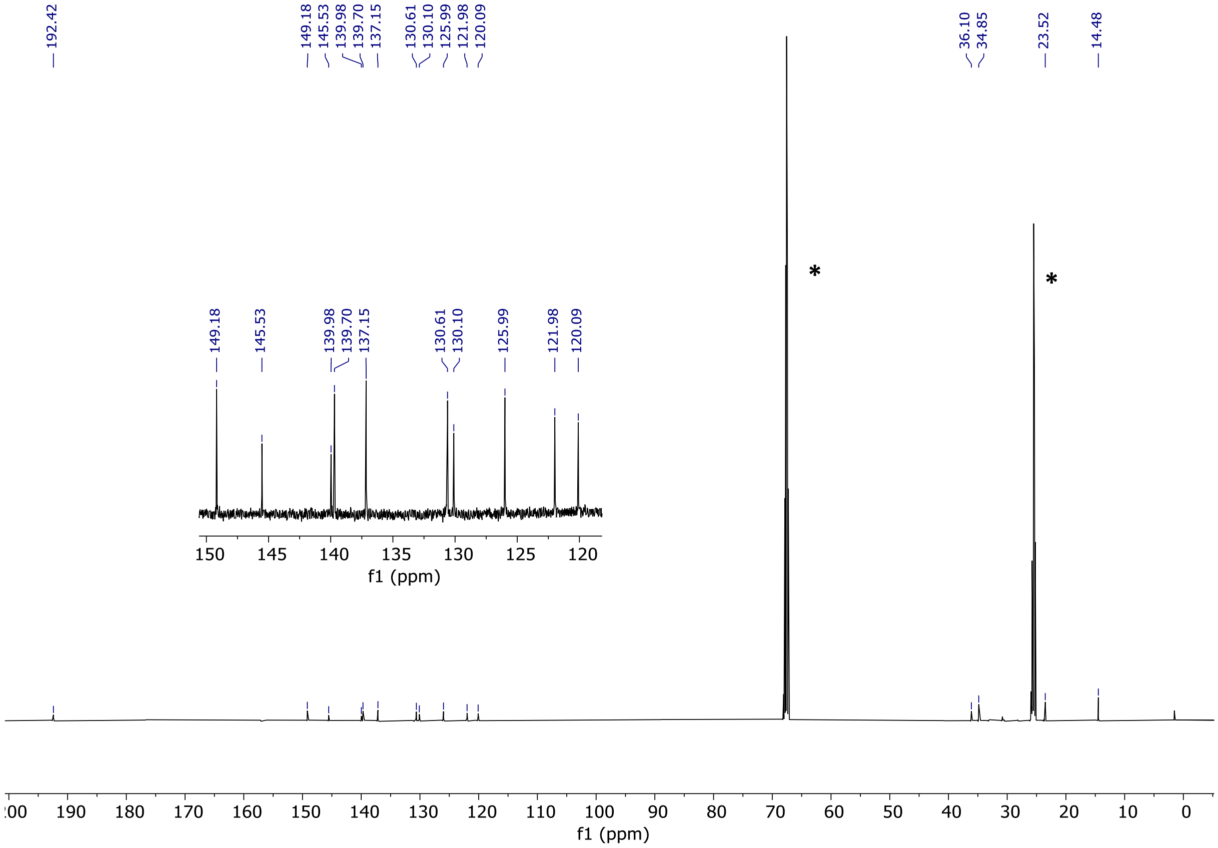

Figure S5. ^{1}H (top) and $^{13}C\{^{1}H\}$ (bottom) NMR spectra (d_8-THF) of $\mathbf{2_{6N}}$ (* = residual solvent).

2$_{7N}$:

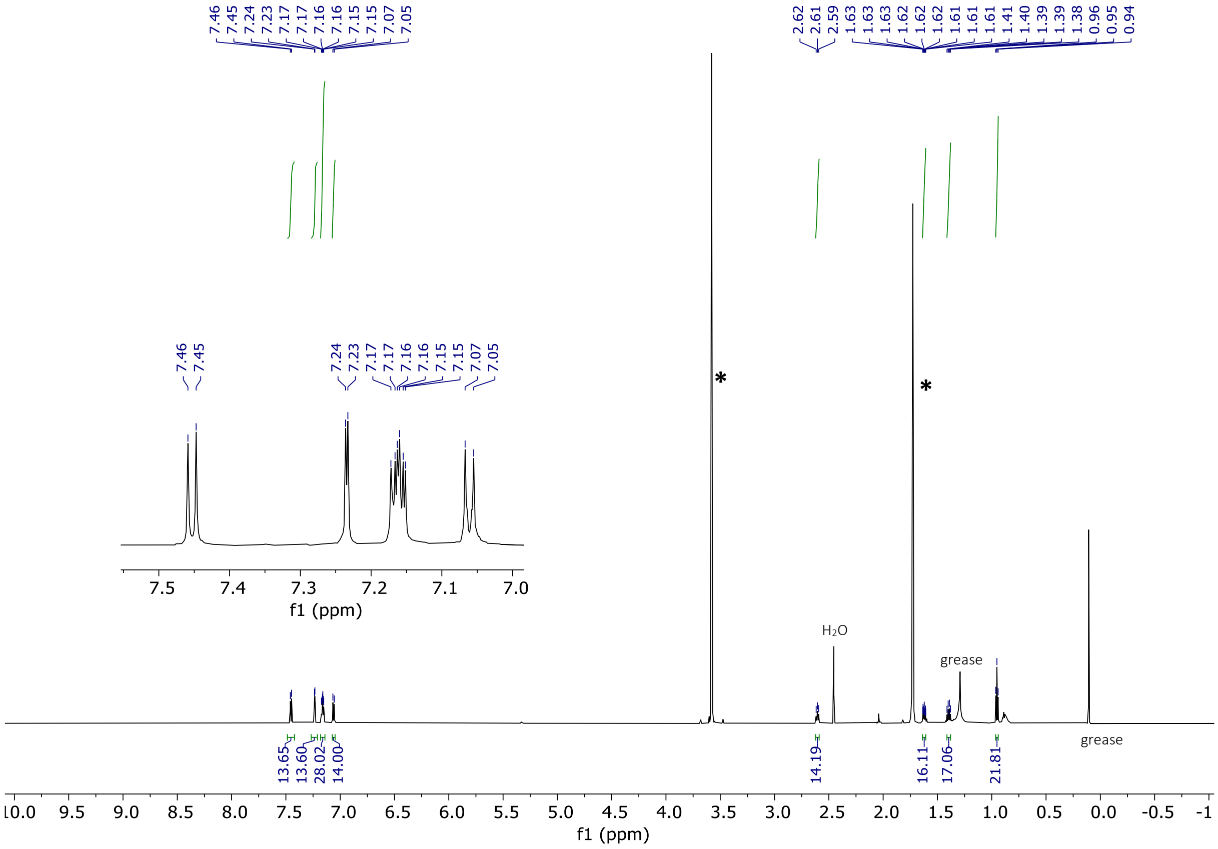

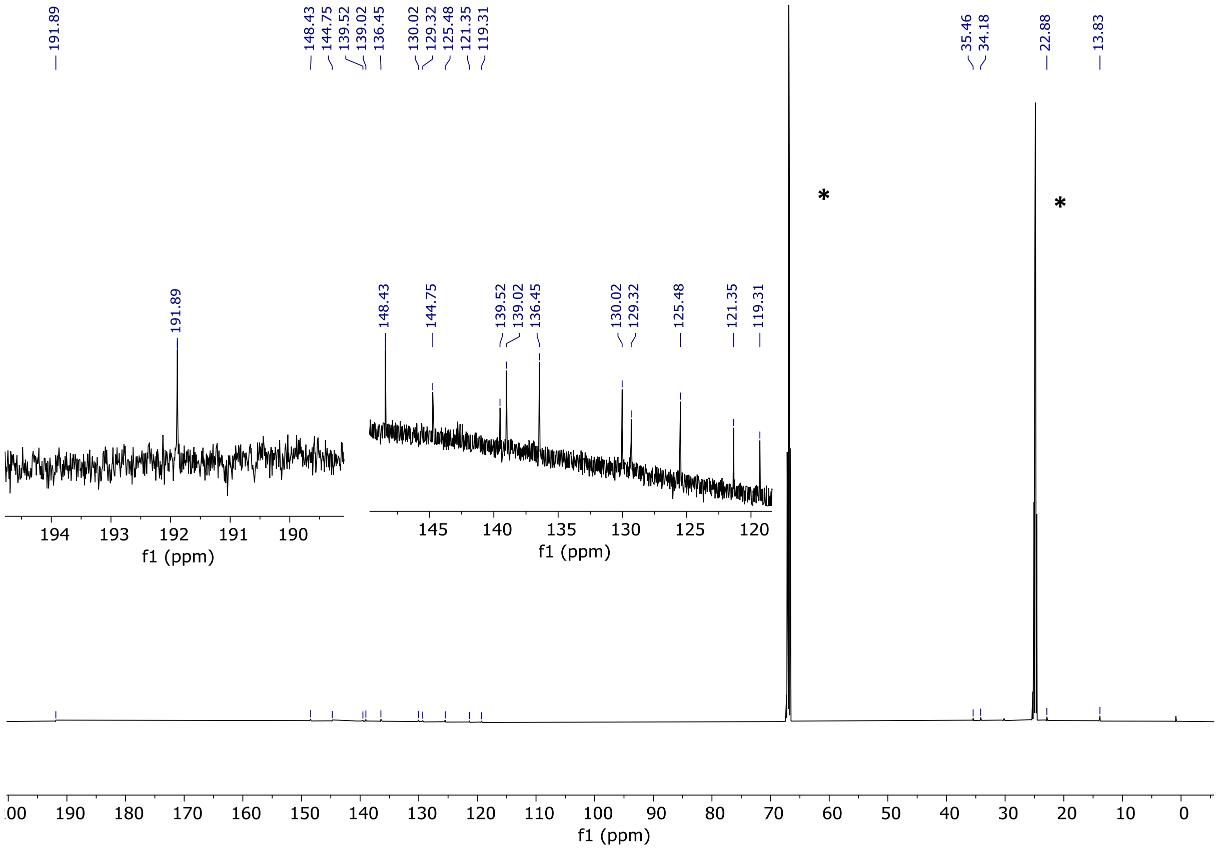

Figure S6. ^{1}H (top) and $^{13}C\{^{1}H\}$ (bottom) NMR spectra (d_8-THF) of **2$_{7N}$** (* = residual solvent).

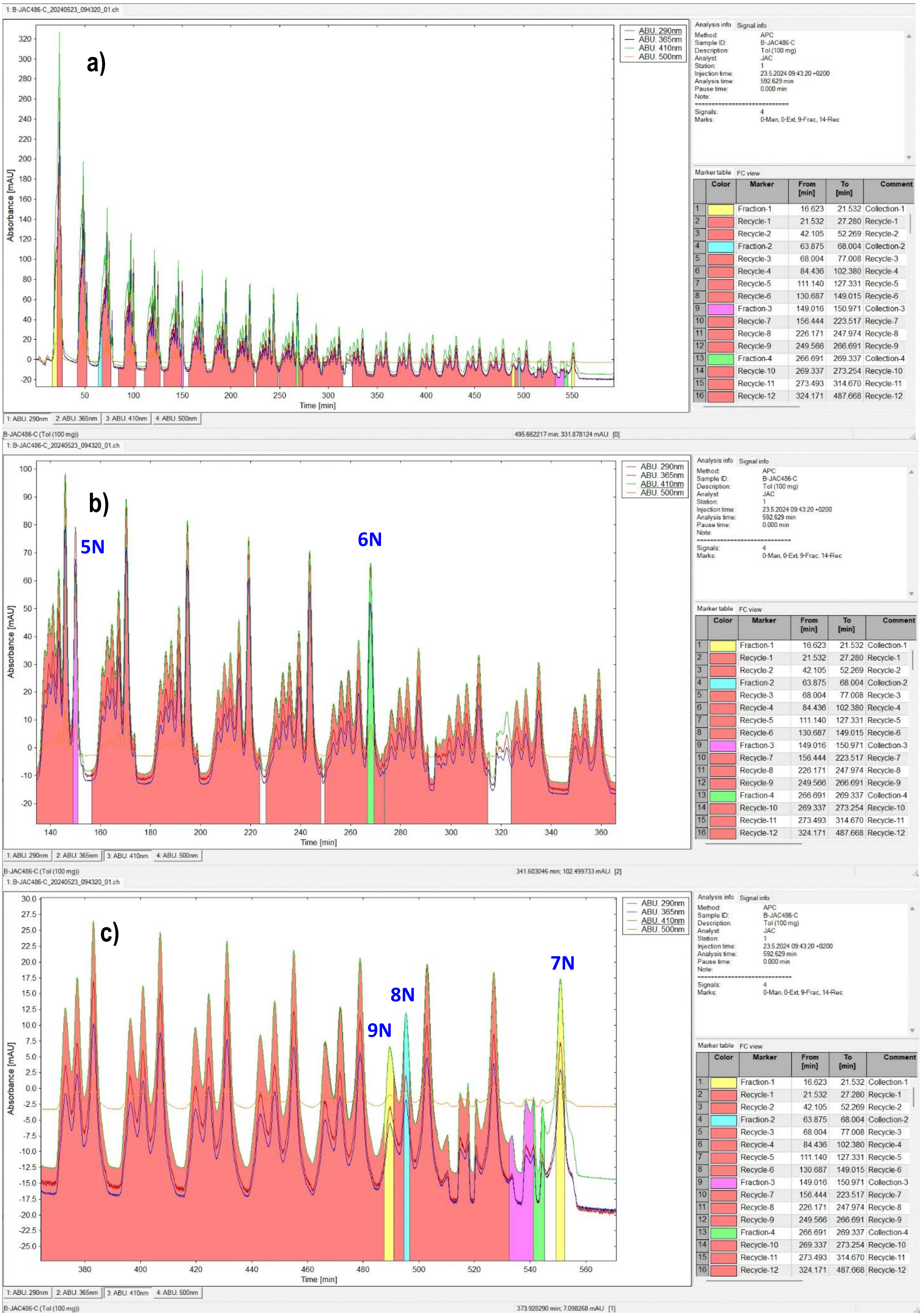

Figure S7. Preparative recycling GPC chromatogram of **2** purification. a) Elution/recycling times (whole run), and b)-c) zoom at the 135-365/365-580 min interval, respectively. Collected fractions indicated by "mN" sizes.

4. X-Ray crystallography

Table S1. XRD Crystal data and structure refinement for **M2**.

Identification code	JAC424_CCDC
CCDC number	2389423
Empirical formula	$C_{23}H_{20}BrNO$
Formula weight	406.31
Temperature/K	100
Crystal system	monoclinic
Space group	$P2_1$
a/Å	15.4795(9)
b/Å	41.8239(17)
c/Å	5.5257(3)
α/°	90
β/°	89.992(5)
γ/°	90
Volume/Å^3	3577.4(3)
Z	8
ρ_{calc}g/cm^3	1.509
μ/mm^{-1}	2.310
F(000)	1664.0
Crystal size/mm^3	0.28 × 0.12 × 0.03
Radiation	Mo Kα (λ = 0.71073)
2Θ range for data collection/°	4.702 to 52.044
Index ranges	$-19 \leq h \leq 19$, $-51 \leq k \leq 51$, $-6 \leq l \leq 6$
Reflections collected	40985
Independent reflections	12610 [R_{int} = 0.0821, R_{sigma} = 0.1384]
Data/restraints/parameters	12610/1115/941
Goodness-of-fit on F^2	1.003
Final R indexes [I>=2σ (I)]	R_1 = 0.0666, wR_2 = 0.1585
Final R indexes [all data]	R_1 = 0.1586, wR_2 = 0.2197
Largest diff. peak/hole / e Å^{-3}	1.16/-1.77
Flack parameter	0.34(3)

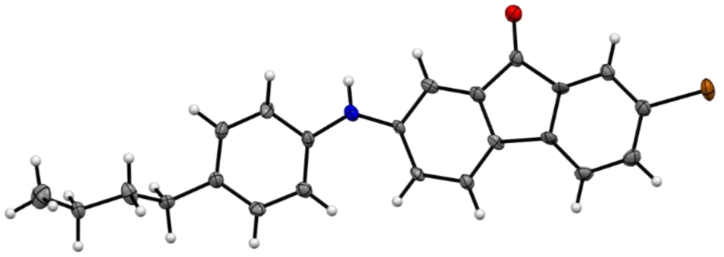

Figure S8. ORTEP representation (50% probability ellipsoids) of the X-ray crystal structure for **M2** (JAC424_CCDC).

5. References

1. Ayuso-Carrillo, J.; Fina, F.; Galleposo, E. C.; Ferreira, R. R.; Mondal, P. K.; Ward, B. D.; Bonifazi, D. One-Step Catalyst-Transfer Macrocyclization: Expanding the Chemical Space of Azaparacyclophanes. *J. Am. Chem. Soc.* **2024**, *146* (24), 16440-16457.
